# Photonic (computational) memories: tunable nanophotonics for data storage and computing

**DOI:** 10.1515/nanoph-2022-0089

**Published:** 2022-05-16

**Authors:** Chuanyu Lian, Christos Vagionas, Theonitsa Alexoudi, Nikos Pleros, Nathan Youngblood, Carlos Ríos

**Affiliations:** Department of Materials Science & Engineering, University of Maryland, College Park, MD, USA; Institute for Research in Electronics and Applied Physics, University of Maryland, College Park, MD, USA; Department of Informatics, Aristotle University of Thessaloniki, Thessaloniki, Greece; Center for Interdisciplinary Research and Innovation, Aristotle University of Thessaloniki, Thessaloniki, Greece; Department of Electrical and Computer Engineering, University of Pittsburgh, Pittsburgh, PA, USA

**Keywords:** optoelectronics, photonic computing, photonic memory, tunable nanophotonics

## Abstract

The exponential growth of information stored in data centers and computational power required for various data-intensive applications, such as deep learning and AI, call for new strategies to improve or move beyond the traditional von Neumann architecture. Recent achievements in information storage and computation in the optical domain, enabling energy-efficient, fast, and high-bandwidth data processing, show great potential for photonics to overcome the von Neumann bottleneck and reduce the energy wasted to Joule heating. Optically readable memories are fundamental in this process, and while light-based storage has traditionally (and commercially) employed free-space optics, recent developments in photonic integrated circuits (PICs) and optical nano-materials have opened the doors to new opportunities on-chip. Photonic memories have yet to rival their electronic digital counterparts in storage density; however, their inherent analog nature and ultrahigh bandwidth make them ideal for unconventional computing strategies. Here, we review emerging nanophotonic devices that possess memory capabilities by elaborating on their tunable mechanisms and evaluating them in terms of scalability and device performance. Moreover, we discuss the progress on large-scale architectures for photonic memory arrays and optical computing primarily based on memory performance.

## 1 Introduction

Information storage has been crucial in humankind’s path towards advanced knowledge and technology. From petroglyphs, through paper, to semiconductor transistors, one task has been common: storing information for perpetuity [1, 2]. The fast development of semiconductors and magnetic recording mediums in the last century and the switch from an analog to a digital world allowed us to reach storage densities now exceeding 10 Gb in a square millimeter [3]. This switch has also enabled capabilities beyond storage, such as the ability to process the data through arithmetic and logic operations at frequencies over a gigahertz [4–6]. The continuing development of both recording media and processing transistors-based systems, thanks to the progress in semiconductors and nanofabrication, has led to devices capable of computing digitally, efficiently, and portably with exceptional success. Current technology, however, is based on Von Neumann’s proposal of a digital computing machine with a dichotomous architecture where data is stored and processed in two different units. The fast development of each separate unit has come with the challenge of moving a large volume of data back and forth between them, generating a bottleneck in the communication channel that stores and fetches data. Several solutions have been proposed to alleviate this so-called von Neumann bottleneck, of which the use of fiber optic interconnects is the most commonly used in today’s data centers. This solution uses light to move data back and forth at the maximum speed available in nature. However, despite the rapid progress in optical interconnects reaching shorter and shorter distances [7], [8], [9], [10] and recently penetrating on-board communications and DRAM-CPU photonic bus interconnects [11], [12], [13], the processor and the memory banks within data centers still operate with electrical signals. Thus, electro-optical conversion is required at both ends, with an energy consumption penalty. Although on-chip optical links have been proposed by cointegrating photonics with silicon nanoelectronics [14, 15] and scalable 3D silicon photonic–electronic integrated circuits are becoming a reality [16], the energy consumption of electro-optical conversions still poses a challenge. This challenge becomes prominent with the increasing computing demands of modern data centers. With their fast-growing capacity, they face heat dissipation challenges both on- and off-chip through charging/discharging interconnects; thus, requiring sophisticated and energy-hungry refrigeration systems [17], [18], [19]. A solution to speed and heat dissipation problems is an architecture in which the memory elements can be read optically, thus avoiding one electro-optical conversion and undesired Joule heating. Another alternative is to perform computational tasks directly in memory, thus reducing data movement and mimicking the computational capabilities of the brain. The processing and memory units will be colocated, leading to non-von Neumann architectures that eliminate the memory access bottleneck. To achieve these two solutions, the quest for photonic memories is paramount, especially those with multilevel capabilities that are crucial in analog brain-inspired architectures.

Here, we review the state-of-the-art photonic memories, focusing on rewritable mechanisms integrated into classical computing architectures. We start by studying devices and move to architectures following the structure:

– Section 2 overviews the physical phenomena and material platforms for data storage with optical readout, whether done in free-space, on-chip, or optical fibers.
– Section 3 covers a compendium of architectures used to grant access to optical memory-cells, i.e. random memory-addressing or content-based addressing, and exploiting the WDM principles to scale towards multi-bit 2D memory-arrays.
– Section 4 discusses the optical architectures for digital, in-memory, and neuromorphic computing, emphasizing the optical memories that enable them, aiming to provide an overview of emerging computing application scenarios that may benefit from photonic memory arrays, and highlights a complete roadmap for integrating tunable nanophotonics for both data storage and computing environments.
– Section 5 is an outlook discussing the potential applications photonic memories could enable outstanding challenges for photonic memories, and research opportunities moving forward.

## 2 Photonic memories

**Figure 1:**
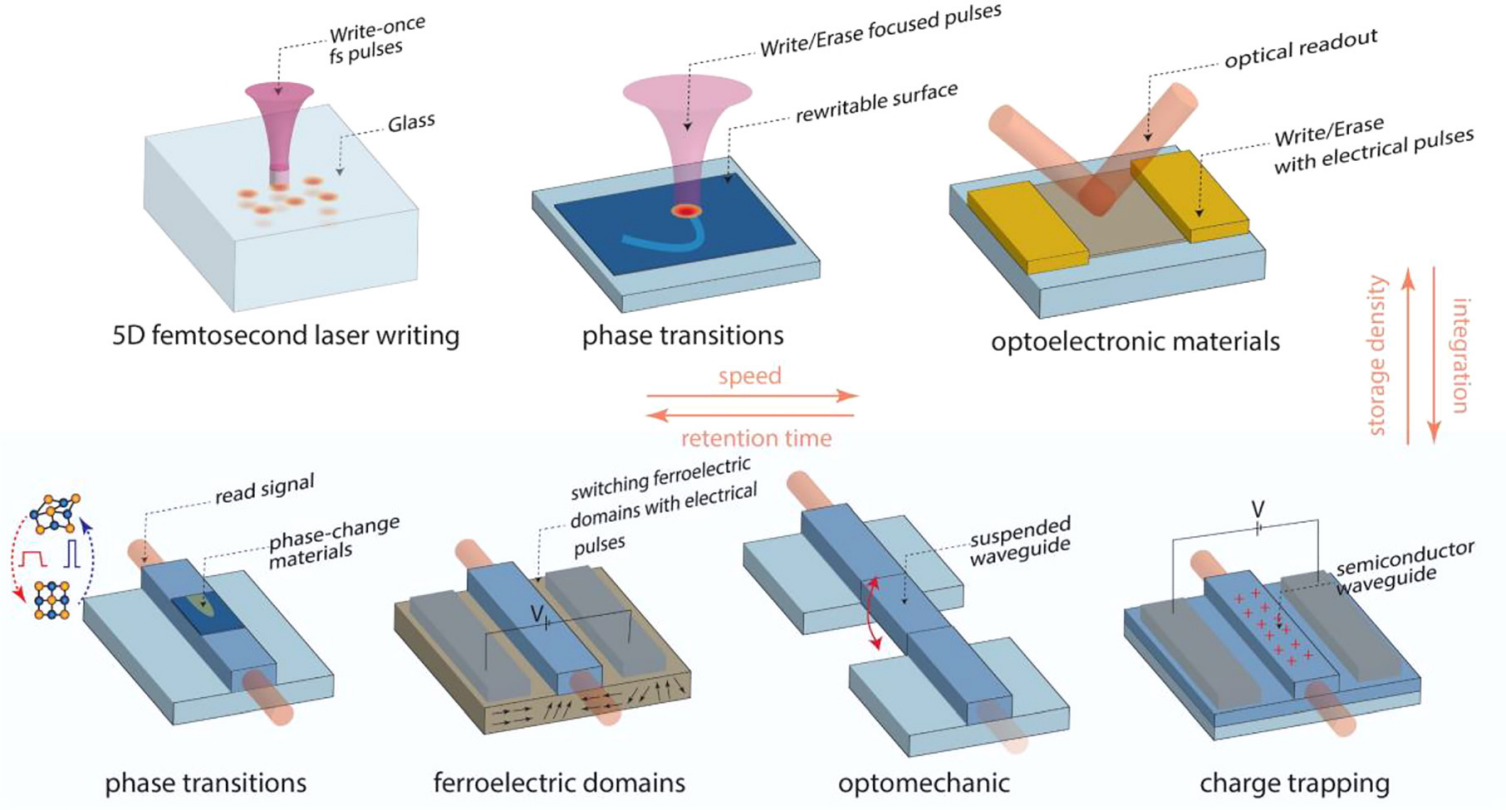
Photonic memory platforms. Top: free-space high-volume data storage. Bottom: photonic integrated circuit approaches for rewritable data storage.

The lack of interaction between photons given their bosonic nature, except under controlled quantum-level conditions [20], means that any light-based memory relies on light–matter interactions to store information [21, 22]. The quest to find mechanisms to write, erase, and read information using optical beams becomes a synergetic effort between finding optimized optical platforms together with suitable material properties and physical phenomena. Photonic memories can be categorized in several ways, depending on the material mechanism, the nature of the optical measurement, or simply the performance metric. They can be volatile or nonvolatile, referring to whether the data is stored permanently without the need for power consumption or not, usually also comprising their speed: volatile memories are used in fast accessing protocols, while nonvolatile memories are used to store information permanently, therefore, not facing stringent speed constrains [23]. Memories can also be rewritable or read-only depending on whether the data is stored in a controllable property of a material or engraved following a write-once process. Photonic memories can, in addition, operate using either phase or amplitude modulation of light on integrated or free-space setups. In Figure 1, we summarize the most advanced photonic platforms for data storage following some of these categories.

The following sections will focus on rewritable mechanisms for memory devices that can be integrated into computing architectures. The analog response of some of these mechanisms enables a computational aspect to these optical memories – which we will refer to as computational memories. Although out of the scope of this review, we acknowledge the important progress in high-density optical storage achieved with technologies such as Blue-Ray, the most advanced representative of the compact disks family [24], or, more recently, engraving nanostructures in silica with 5 degrees (5D) of freedom of a light beam to achieve an impressive ∼500 Tb/disk capacity proposed by Sakakura et al. [25], among several other optoelectronic platforms [21, 26].

### 2.1 Phase transition

Transitions between different crystallographic states of specific materials such as chalcogenide phase-change materials, perovskites and transition metal oxides lead to strong modulation of their optical properties, which can, in turn, be harnessed to modulate either (or both) the phase and amplitude of light. In addition, some of these materials display a nonvolatile modulation of their optical properties, which is beneficial for long-term data storage. These properties make optical materials displaying phase transitions ideal for photonic memories, especially those integrated into CMOS processes.

#### 2.1.1 Chalcogenide phase-change materials

Phase change materials (PCMs) are chalcogenides that can repeatedly switch between their amorphous and crystalline states on the order of pico- and nano-second timescales [27, 28]. These materials show a pronounced contrast in electrical resistivity and optical reflectivity over a broad spectral range upon phase transformation [29], [30], [31]. Data can be stored in the phase state of PCMs and retained for decades at room temperature [24]. Since Ovshinsky first proposed the use of PCMs in non-volatile storage technology [32, 33], many works have attempted to program PCMs both electrically (PC-RAM) and optically (optical disks) [34–36]. This section focuses on configuring PCMs to realize modifications in the optical response of a thin film or device.

##### 2.1.1.1 Free space

**Figure 2:**
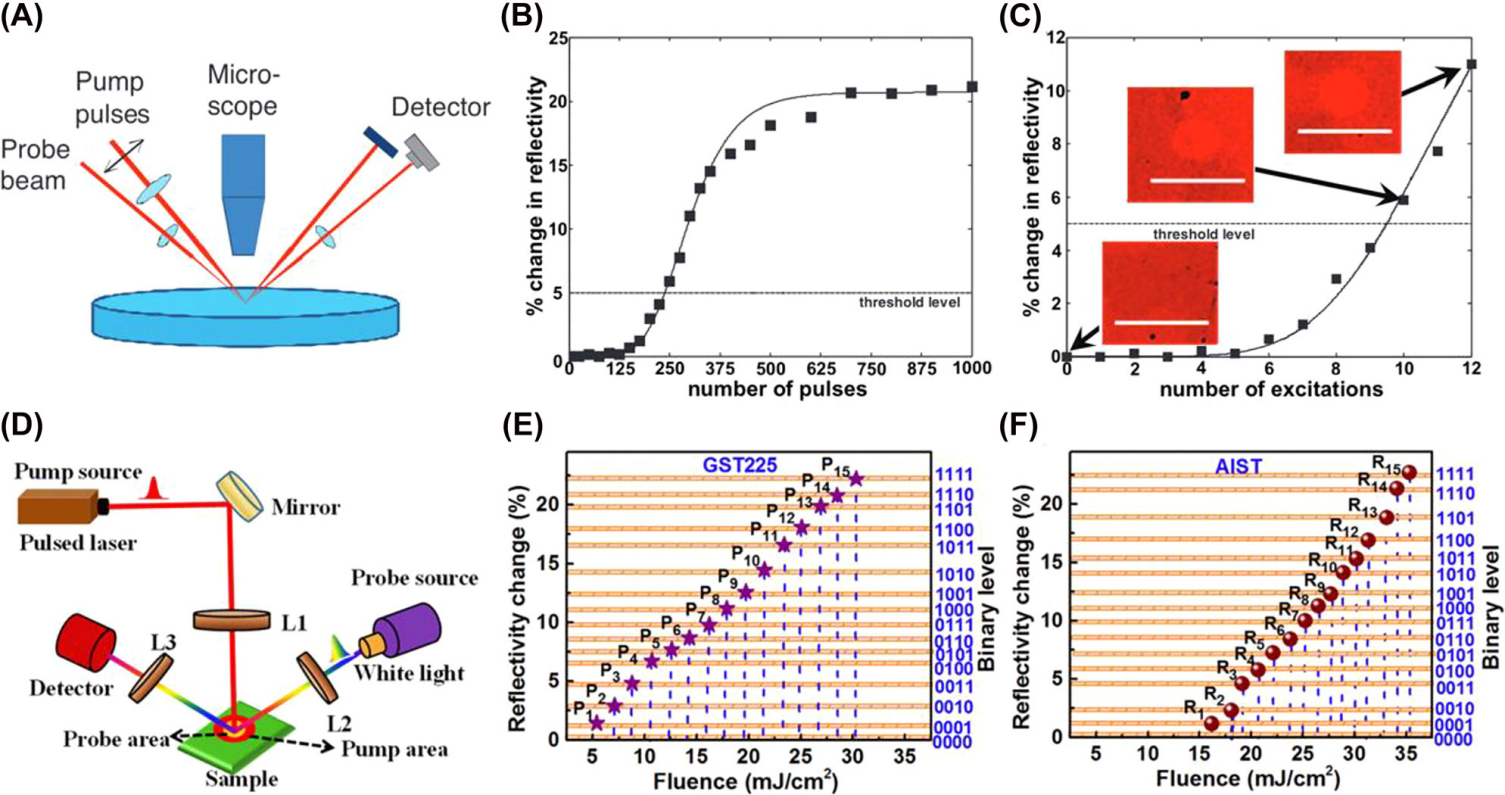
(A–C) Experimental demonstration of the natural accumulation property in PCMs [37]. (A) Schematic of the pump-probe setup for reflectivity measurement. (B) Percent change in GST reflectivity as a function of the number of 85 fs, 3.61 mJ/cm^2^ pulses applied. (C) Percent change in GST reflectivity as a function of the number of excitation events. The inset optical micrographs show the evolution of the laser irradiation mark. (D–F) Realization of 4-bit memory in GST225 and AIST [44]. (D) Optical configuration of the pump-probe lasers. This setup allows optimization of the pump beam diameter to achieve 16 distinctive reflectivity levels in (E) GST225 and (F) AIST.

Phase-change materials have a strong potential for applications in biologically-inspired computing because they exhibit a natural accumulation property (see Section 4.3.1). A single excitation event, either electrical or optical, induces partial crystallization in the PCM, whereas a succession of such events achieves complete crystallization. This property strongly resembles the “integrate and fire” neurons and synapses. Wright et al. were one of the first groups to experimentally demonstrate the four basic arithmetic processes (addition, multiplication, division, and subtraction) in the free-space optical domain using a pump-probe setup shown in Figure 2A [37]. Their experiments fully exploited the natural accumulation and threshold properties of Ge_2_Sb_2_Te_5_ (GST) and became a reference to on-chip integration in future works. Multilevel switching in various PCMs has been studied as a slight change in composition that can give rise to unique material properties [38–42]. Arjunan et al. studied nucleation-dominated GST and growth-dominated AIST to explore the upper limit of storage density in PCMs [43, 44]. By carefully manipulating the power (fluence) of each pulse, they successfully attained a maximum reflectivity contrast of 22.2 and 22.7% between the crystalline and amorphous phases of GST and AIST, respectively. Using this reflectivity window, Arjunan et al. realized 4-bit storage, or 16 distinct reflectivity levels, in both materials at the operating wavelength of 532 nm (see Figure 2E and F). The authors suggest that along with the wavelength division multiplexing technology, multilevel optical phase-change memory devices could easily outperform their electrical counterparts in terms of storage density. Another mechanism to achieve multilevel response below the diffraction-limit is patterning PCM thin films using scanning probe microscopy [45–48]. This technology allows for impressive high densities, although it faces scalability challenges due to the complexity of the write/erase process and the difficult optical readout. The smallest demonstrated feature is as small as 50 nm [45].

##### 2.1.1.2 Fiber optics

**Figure 3:**
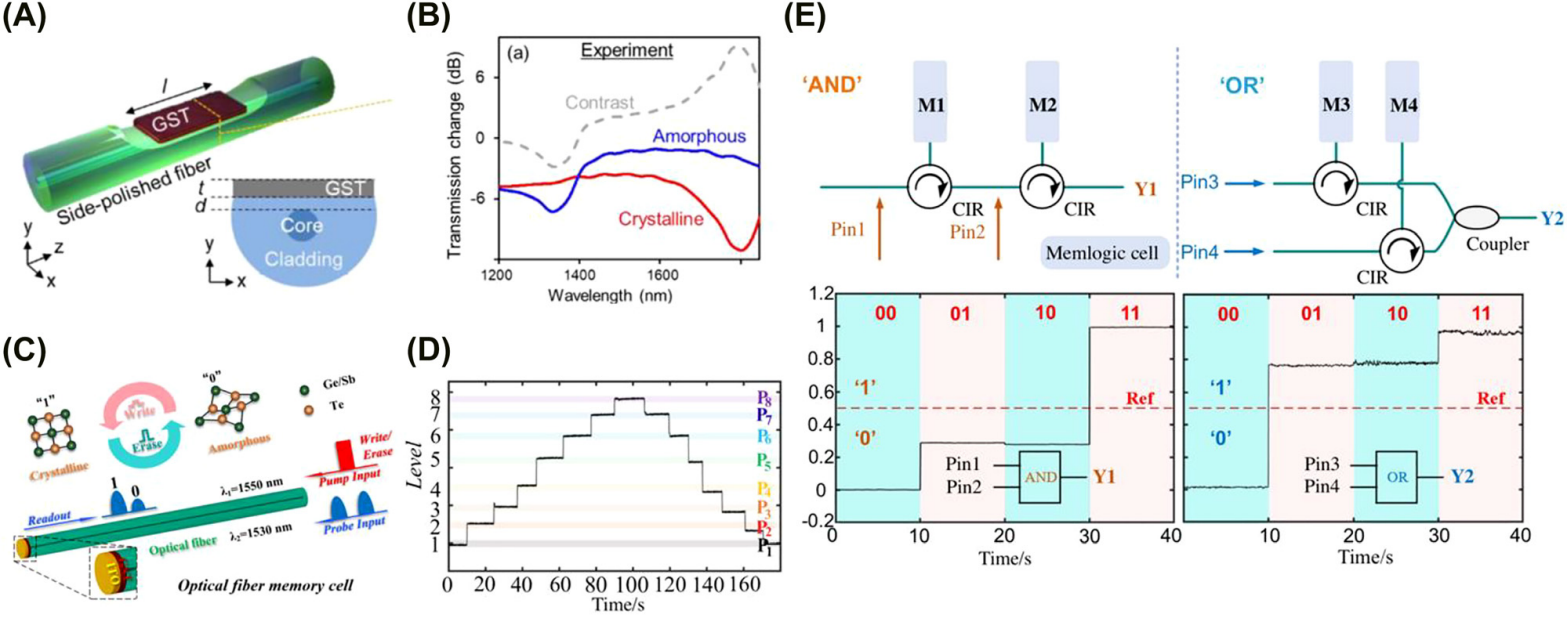
(A) and (B) PCM integration in side-polished optical fiber [49]. (A) Schematic of a PCM integrated optical fiber device capable of reconfigurable transmission attenuation. (B) Experimentally measured spectral transmission change resulted from amorphous (blue line) and crystalline (red) GST modulations. (C–E) PCM integration in end-polished optical fiber [50]. (C) Operation principle of the PCM-based all-fiber memory device. Amorphous GST, the low-reflectivity state, stores logic state 0 while crystalline GST, the high-reflectivity state, stores logic state 1. (D) Experimental demonstration of eight storage levels reached sequentially. (E) Experimental demonstration of logic operations. Left: “AND” logic performed is performed by connecting to cells in series. Right: “OR” logic is performed by placing two cells in parallel and the two outputs are combined with a coupler.

In addition to free space and on-chip integrations, reports have also attempted to integrate phase-change materials in fiber optics to realize optical memory. Figure 3A schematically shows the geometry of the fiber device designed by Martins et al. [49]. A single-mode silica optical fiber is side-polished and a 500 nm GST thin-film is subsequently deposited onto the polished surface. The reduced cladding thickness over the polished region allows evanescent coupling between the guided mode and GST, thereby attenuating the propagating light. Martins et al. reported a 2.8 dB change in absolute transmission at a resonance wavelength of 1315 nm and 9.1 dB at a resonance wavelength of 1708 nm for amorphous phase and crystalline phase GST, respectively (Figure 3B). Numerical simulations suggest that the modulation depth strongly depends on the proximity between the GST thin-film and fiber core, while the position of the resonance wavelength can be adjusted by changing the GST film thickness. Any resonance ranging from 1250 to 1590 nm can be easily obtained using this method. However, this study did not demonstrate reversible phase switching over many cycles because the GST film was not encapsulated against thermally induced deformation and oxidation. To overcome this challenge, Liu et al. designed a PCM-based all-fiber memory device capable of both storage and logic computing functions [50]. The operation principle and device design are shown in Figure 3C. A layer of 120 nm thick GST and a layer of 30 nm ITO are deposited to one end face of a single-mode optical fiber. By modulating the amplitude of the pump pulse, the degree of amorphization (or crystallization) and thus the reflectivity of the GST layer can be effectively controlled. As shown in Figure 3D, Liu et al. demonstrated experimentally 8-level data storage (3-bit) with a maximum optical contrast of 38%. All levels can be accessed from any other level in random order. They further achieved “AND” and “OR” logic operations (Figure 3E) by placing two memory cells in series and parallel, respectively. This introduces a new perspective for developing complex, intelligent fiber systems in the future.

##### 2.1.1.3 Integrated photonics

**Figure 4:**
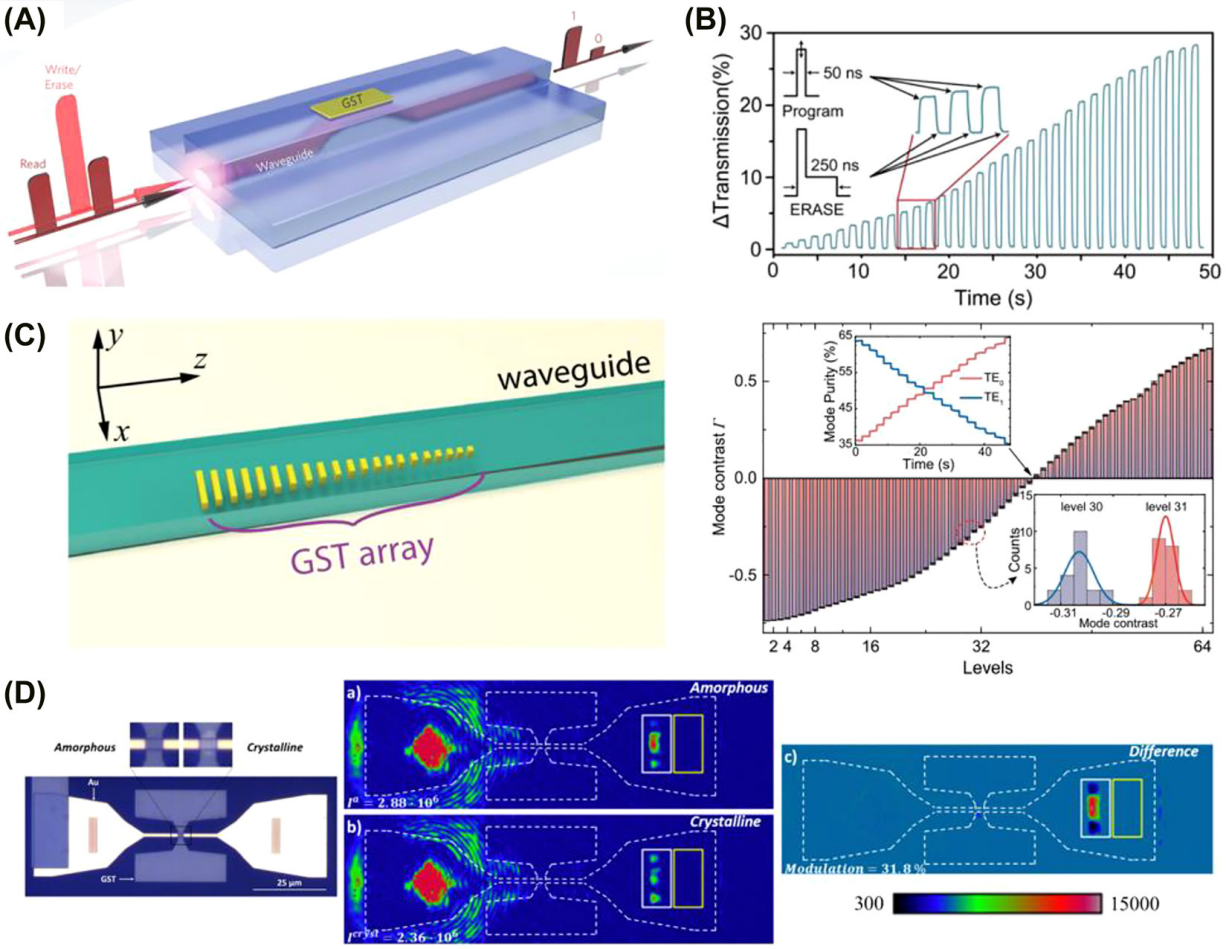
All-optical waveguide integrated and plasmonic PCM-based devices. (A) Device design and operation principle of an all-photonic nonvolatile memory device showing the evanescent coupling between the guided light and GST [53]. (B) Measurement of multilevel operation demonstrating over 5 bits storage capabilities [54]. (C) A multilevel programmable phase-change metasurface mode converter. Left: 3D rendering of the phase-gradient metasurface array made of GST. Right: 64 distinct levels of mode contrast corresponding to 6-bit storage accurately controlled by the mode converter [55]. (D) PCM integration in surface plasmon waveguides. Left: Optical micrograph of the plasmonic switch [60]. Middle: Intensity images showing the scattered and transmitted light when GST is (a) amorphous and (b) crystalline. Right: Contrast in the output intensity is highlighted taking the difference between (a) and (b). The PCM switching was triggered with an external laser source.

For the on-chip integration of PCMs, the literature reports phase transformations triggered both by photothermal and electrothermal switching. Pernice and Bhaskaran were the first to propose a fully-integrated all-photonic memory by embedding PCMs onto nanophotonic waveguides and realizing both the switching and readout with guided modes [51, 52]. This was first experimentally demonstrated using GST225. Phase transformation in GST can be triggered entirely in the optical domain by exploiting the nonzero complex refractive index of GST. Through the evanescent coupling between light traveling inside the waveguide and GST, Rios et al. realized that integrated photonic devices could be switched and read in the optical domain [53]. Figure 4A shows the design and operation principle of the device. Since crystalline GST (c-GST) displays a larger extinction coefficient and thus is more absorptive than amorphous GST (a-GST), c-GST causes a more significant attenuation of light travelling inside the waveguide. On the other hand, a-GST is less absorptive and allows more light to be transmitted. This effectively leads to a transmission contrast and allows data to be stored in the two phases. The absorbed light is converted to heat, and if the GST film is heated above its transition temperature, then either the crystallization or the amorphization process will be triggered (depending on the pulse shape). Using this bistable property of GST, Rios et al. achieved a total transmission contrast of 21% in a 5 µm GST device. Eight individually addressable storage levels in a single memory cell, corresponding to 3-bit storage, were demonstrated in a single memory cell [53]. In a following study (Figure 4B), 5-bit storage with 34 nonvolatile levels was achieved using a single double-step optical pulse instead of a train of pulses that could fully crystalize the cell regardless of its previous state [54]. This was later improved to 6-bits of storage, shown in Figure 4C, using subwavelength patterning of reconfigurable GST metasurfaces [55]. Other less scalable approaches for integrated photonics have been proposed using external laser switching [56–59].

It has also been shown that GST can be integrated into plasmonic-based photonic circuits to control the propagation of surface plasmon polaritons (SSP) all in the optical domain. Rude et al. demonstrated that the transmitted intensity of the plasmonic mode can be controlled by switching from the low-loss amorphous phase to the high-loss crystalline phase [60]. Using the device shown in Figure 4D, an overlap area of $2\times 5\;\mu {\text{m}}^{2}$ between an 80 nm thick GST film and the SPP waveguide can result in a 31% total transmittance decrease as the GST is switched from amorphous to the crystalline phase. This design supports GST phase transformation both by applying electrical pulses or optical pulses through an externally controlled laser.

**Figure 5:**
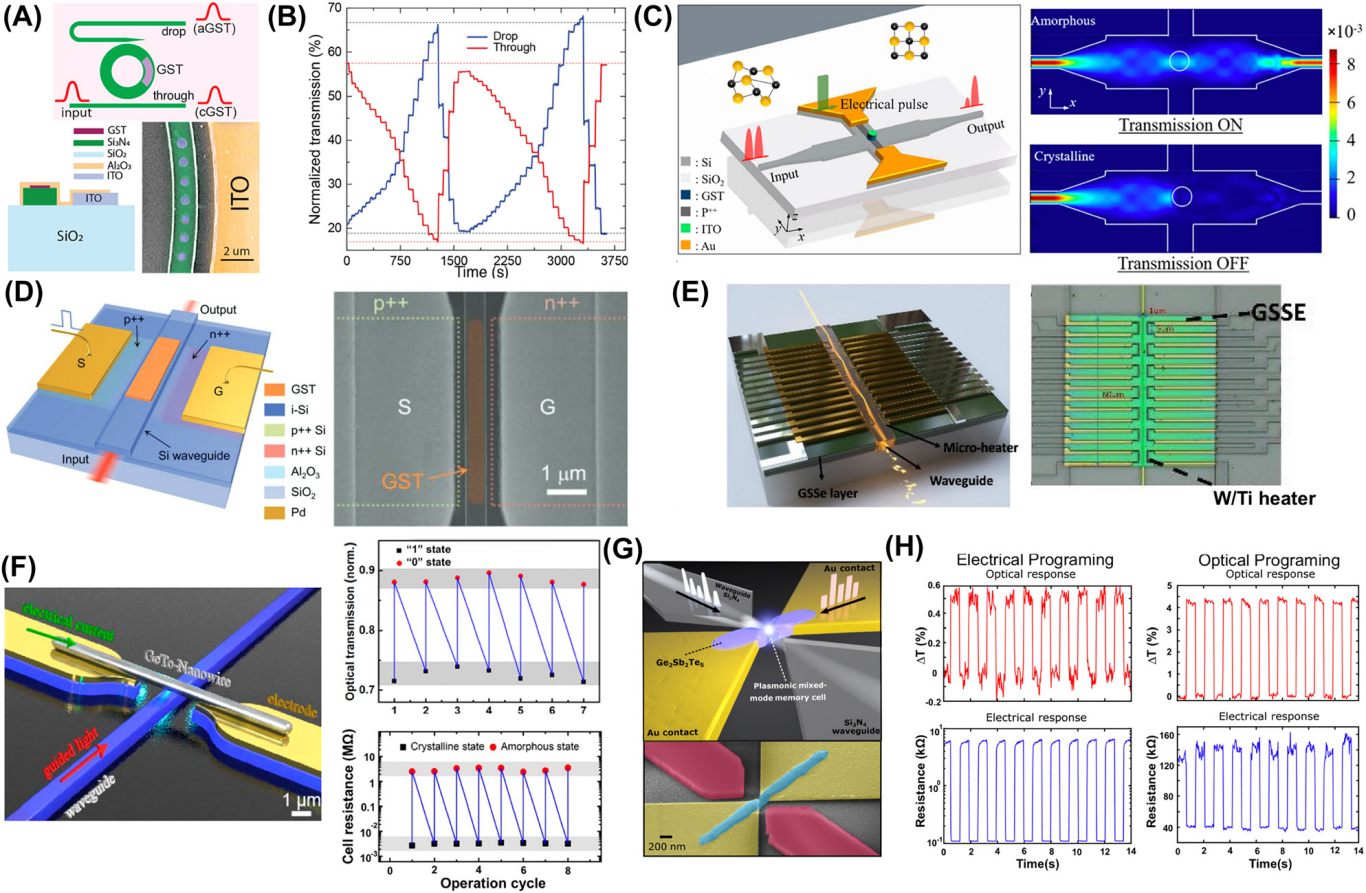
Electrothermal and mixed-mode switching of PCM-integrated memory devices. (A) and (B) Low-loss 1×2 optical switch by integrating patterned GST to a ring resonator [64]. (A) Top: Operation principle of the 1×2 optical switch. Bottom left: Cross-sectional view of the device structure. Bottom right: False colored top-view SEM image of the subwavelength sized GST disks. (B) Real-time transmission measurement during two full cycles of switching. (C) Demonstration of an electrically driven optical memristive switch based on GST integrated MMI [66]. Left: Schematic of the device design. GST disk is encapsulated and placed at the center of the MMI. Right: FDTD simulations showing the power distribution as light propagates through the MMI when the GST disk is amorphous (Top) and crystalline (bottom). (D) Device design of an electrically reconfigurable photonic switch using a PIN diode heater [67]. (E) Optical image showing paired W/Ti heaters placed in series along both sides of the waveguide to electrothermally switch GSSe and realize 4 × 1-bit memory [68]. (F) Demonstration of mixed-mode read-out in GeTe nanowire embedded optoelectronic circuits [69]. Left: Illustration of device design. Optical transmission contrast (Top Right) and electrical resistance contrast (Bottom Right) both realized in the optical domain. (G) and (H) Plasmonic nanogap enhanced GST-based memory cell [70]. (G) Schematic of the device zoomed in at the nanogap. (H) Device’s optical and electrical response under electrical programing (Left) and optical programing (Right).

Alternatively, electrothermal switching using external resistive heaters allows the PCM to be electrically isolated and switched indirectly. This sets limitations to the heater material as it needs to be both conductive and not absorptive in the near-infrared regime. Examples of transparent resistive heaters are indium–tin-oxide (ITO) [61–64] and fluorine-doped tin-oxide (FTO) [65]. Transparent heaters have the advantage of low losses. Kato et al. demonstrated switching operation by injecting a 100 ns pulse of 20 mA for amorphization and a 100 ms pulse of 12 mA for crystallization of GST225 patches [62]. The optical readout showed an average extinction ratio of 1.2 dB between 1525 and 1625 nm wavelengths. Wu et al. suggested that switching large GST patches can be inefficient and made improvements to the current structure [64]. They employed small Al_2_O_3_ encapsulated GST disks in their 1 × 2 microring resonators to realize uniform heating and prevent GST deformation during the melt-quench process. Additionally, the ITO heater was placed 1.1 μm away from the microring to reduce losses as shown in Figure 5A. This way, the GST disks are not in direct contact with the heater and are instead heated through the Al_2_O_3_ heat conduction layer that encapsulates both the GST disks and the ITO heater. A switching contrast of 5.2 and 6 dB were achieved at the through port and drop port, respectively, using the ITO heater for crystallization and optical pulses for amorphization. The group also attempted all-optical switching and achieved much higher contrasts of 13.5 dB at the through port and 21.3 dB at the drop port. Graphene is another example of a transparent resistive heater due to its unique 2D structure and tunable optical absorption. Simulation and experimental data have proven the feasibility of integrating graphene with PICs and could enable highly efficient electrical switching of optical PCMs [71, 72].

Another way to fabricate on-chip resistive heaters is by p-type and n-type doping of silicon on SOI substrates [67, 71], [72], [73]. A recent study by Zhang et al. showed reliable switching results using uniformly p^++^ doped-silicon heaters in multimode interferometer (MMI) devices [66]. The device design is depicted in Figure 5C. The GST/ITO stack is placed at the center of the MMI where light is focused and can be most effectively attenuated. The doped silicon strip is positioned orthogonally to the direction of the MMI and connected to electrodes at the two ends. By carefully controlling the amplitude and width of the write/erase pulses, partial crystallization of GST was obtained, and five distinct levels were achieved in this device. However, the heavy p-doping of these silicon microheaters required short interaction lengths to minimize optical absorption, leading to high energy consumption (driving voltages > 10 V) and low cyclability (5–50 times). To reduce optical loss and enable more efficient microheaters, Zheng et al. used a waveguide-integrated silicon PIN diode (p-type–intrinsic–n-type junction) heater shown in Figure 5D to switch GST electro-thermally [67]. They achieved over 500 switching cycles using a driving voltage of 1 V for crystallization and 2.5 V for amorphization with almost no losses (0.02 dB $\mu {\text{m}}^{-1}$ ). A similar design has also been extended to *n* ^++^–*n*–*n* ^++^ microheaters integrated into silicon waveguides [74]. Moreover, using metal heaters arranged in a serial manner along the waveguide as shown in Figure 5E have recently demonstrated the reversible switching of Ge_2_Sb_2_Se_5_, a transparent material in amorphous state, on silicon waveguides and up to 0.5 million cycles [68].

Devices introduced until this point only provided a change in optical readout upon excitation. In addition to their optical properties, many PCMs such as GST and GeTe undergo significant changes in electrical resistance upon phase transformation. Theoretically, it is possible to read and write information to PCMs in optical and electrical domains. Nevertheless, these mixed-mode devices are challenging because metallic electrodes must be placed near the PCM to enable electrical switching, and metals are highly lossy. Additionally, a large volume of material needs to be switched to ensure stable readouts in the optical domain instead of the filaments achieved via current switching. To overcome these challenges, Lu et al. fabricated a mixed-mode device (see Figure 5F) by suspending a GeTe nanowire above an on-chip photonic waveguide [69]. The nanowire is supported by metallic contacts at both ends, which allows electrical access, and evanescent coupling with the waveguide below allows optical access to the PCM. Although changes in DC resistance and optical transmission could be observed when the device is under all-optical operation, the two could not be measured simultaneously as higher optical pulse energy is required to achieve resistance contrast than transmission contrast. Nevertheless, a higher energy pulse could only result in a transient response in optical properties. This is likely because the volume of switched material inside the nanowire is insufficient to induce measurable changes in the optical absorption. To increase the volume of PCM switched, the distance between the guided light and the PCM, as well as the distance between the electrodes need to be reduced during optical and electrical operations respectively.

Farmakidis et al. successfully achieve dual electrical-optical functionality by incorporating a plasmonic nanogap in the photonic waveguide, as shown in Figure 5G [70]. Both optical and electrical signals intersect in this plasmonic region. Since the Au contacts serve both as the device electrodes and as the plasmonic waveguide, the volume and separation between the electrical contacts decreases. This effectively strengthens the electrical field within the nanogap and decreases the volume of PCM that needs to be switched to obtain measurable contrasts in optical absorption due to the plasmonic confinement of the optical mode. All-optical operation also benefits from the device design as the reduction in mode volume in the nanogap can improve light–matter interaction. Simultaneous changes in both resistance and transmission of the device during optical programming and electrical programming are plotted in Figure 5H. The readout has a higher contrast and lower noise when the device is read the same way it is programmed, while the readout signal has less contrast when the device is read in the complementary domain. This is attributed to the difference in the overlap between the electric field used for programming and the optical plasmonic mode, which requires further optimization.

#### 2.1.2 Perovskites

**Figure 6:**
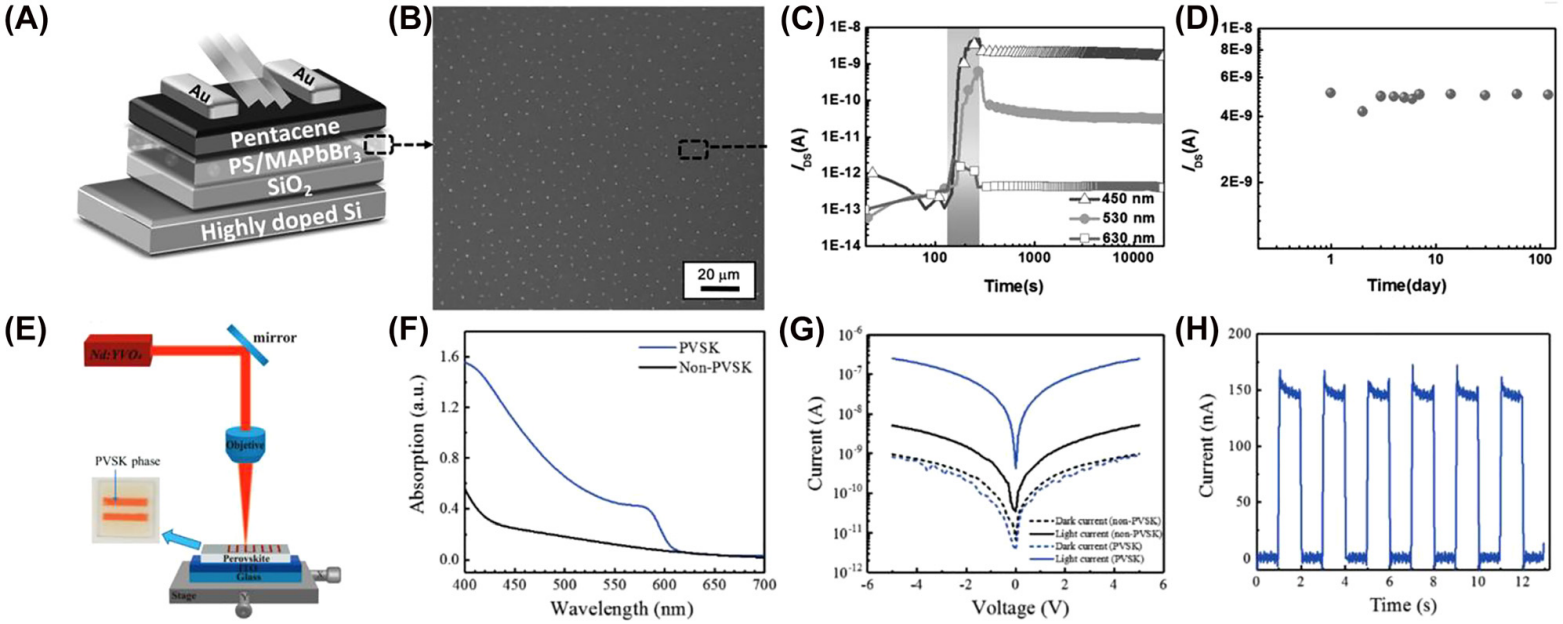
Perovskite-based photomemory devices. (A–D) An organic–inorganic hybrid perovskite-based photomemory device that exhibits multilevel memory behavior [76]. (A) Structural schematic of the bottom-gate, top-contact device. (B) Zoomed in SEM image of the MAPbBr_3_/PS composite film. (C) The device’s distinctive current response to excitation wavelengths 450 , 530, and 630 nm. (D) Long and stable retention for 120 days. (E–H) Photomemory device based on phase-change perovskite CsPbIBr_2_ [77]. (E) Setup for laser direct writing to pattern the non-PVSK phase (transparent in inset) to the PVSK phase (orange in inset). (F) Optical absorption spectrum of the PVSK (blue) and non-PVSK (black) phases. (G) *I*–*V* curves showing device response under dark and white light illumination. (H) Real-time measurement of device switching behavior.

Perovskite materials have been extensively studied over the past few years due to their exceptional optoelectronic properties such as high absorption coefficient, high charge-carrier mobility, and long charge-carrier lifetime [75]. Recent studies have demonstrated that solution-processed organic-inorganic hybrid perovskite materials can exhibit long-term nonvolatile multilevel memory behavior [76]. Chen et al. successfully achieved long-term nonvolatility by adding discrete MAPbBr_3_ nanoparticles into a polystyrene matrix shown in Figure 6B, thereby allowing the photogenerated electrons to be trapped inside the insulating polymer matrix. A large device on/off current ratio was retained for three months (Figure 6D). Moreover, the distinctive current response of the device to various excitation wavelengths enables multilevel memory (see Figure 6C). Nevertheless, the charge trapping mechanism represents a “weak” nonvolatility response since charges might leak and are sensitive to parasitic electrical signals. A more recent study solved this problem by applying an inorganic perovskite material CsPbIBr_2_ that can reversibly switch between its nonperovskite orthorhombic ( δ ) and perovskite cubic phases ( α ) [77]. Using the experiment depicted in Figure 6E, the δ to α phase transformation can be triggered through laser heating while the reverse process can be achieved through moisture exposure. Absorption spectra (see Figure 6F) reveal that the α phase has a much higher optical absorption in the visible spectra than the δ phase. A photocurrent ratio of over 50 was measured under white light illumination between the perovskite and non-perovskite phases (see Figure 6G). This contrast is sufficient to distinguish the “1” state stored in the high photocurrent α phase and the “0” stored in the low photocurrent δ phase.

#### 2.1.3 Transition metal oxides

Transition metal oxides are a unique group of materials that can demonstrate large, nonlinear changes in their optical, electrical, thermal, and magnetic properties when changing their electronic structure [78]. This change can be controlled during material growth (e.g., tuning stoichiometry and oxygen vacancies in WO_*x*_ and TiO_2_) or made reversible as in the case of the insulator-to-metal phase transition (IMT) in VO_2_. This IMT occurs due to distortions in the crystal lattice and strongly correlated electron behaviors, which cause an abrupt physical change in the material from an insulator to a metal. VO_2_ is one of the most commonly studied optical materials in this IMT family owing to its reversible phase transition near room temperature and favorable optical properties with large contrast between the metal and insulating states [79–81]. Several groups have demonstrated on-chip modulation of optical signals and all-optical switching of waveguide-integrated VO_2_ for active photonic devices [80–85].

The IMT process in VO_2_ is most commonly controlled via thermal cycling and is thus volatile in nature. However, other switching approaches, such as ionic gating [86], applying an electric field [87], mechanical strain [88], or thermal biasing [85, 89], could be used to create electrically reconfigurable, nonvolatile photonic memory on-chip. For example, Jung et al. recently demonstrated nonvolatile optically addressable memory at room temperature by integrating VO_2_ on a waveguide while applying a constant biasing voltage [85]. The applied voltage bias brings the VO_2_ within the IMT hysteresis region, allowing an optical pulse to switch the material from its insulating to the metallic state. Since both the optical and electrical properties change during the IMT, this device also enables the conversion of information from the optical to electrical domain by observing both the transmission and resistance changes of VO_2_. The authors also demonstrated nonvolatile IMT oscillations induced by optical pulses in the case of current biased VO_2_ microwires [90]. Interestingly, the devices remained in this nonvolatile metastable state for days, even without any applied electrical or optical signal. Such nonlinear devices could be useful for optoelectronic neuromorphic computing architectures and information processing [91, 92].

### 2.2 Charge trapping effects

The complex refractive index of a semiconductor can be modulated by injecting or removing charges from the region where the optical mode propagates, or a region evanescently coupled to the light. This is achieved by adding doping profiles to silicon, germanium, InP, or other semiconductors, which are, in turn, externally modulated using an electrical signal. Leveraging the CMOS semiconductor fabrication, Photonics has also benefitted from the advances in microelectronics. The result is a variety of photonic integrated devices that store data using charge trapping and electrical gating effects, some of which we describe in this section.

#### 2.2.1 Bistable SOA-based memories

**Figure 7:**
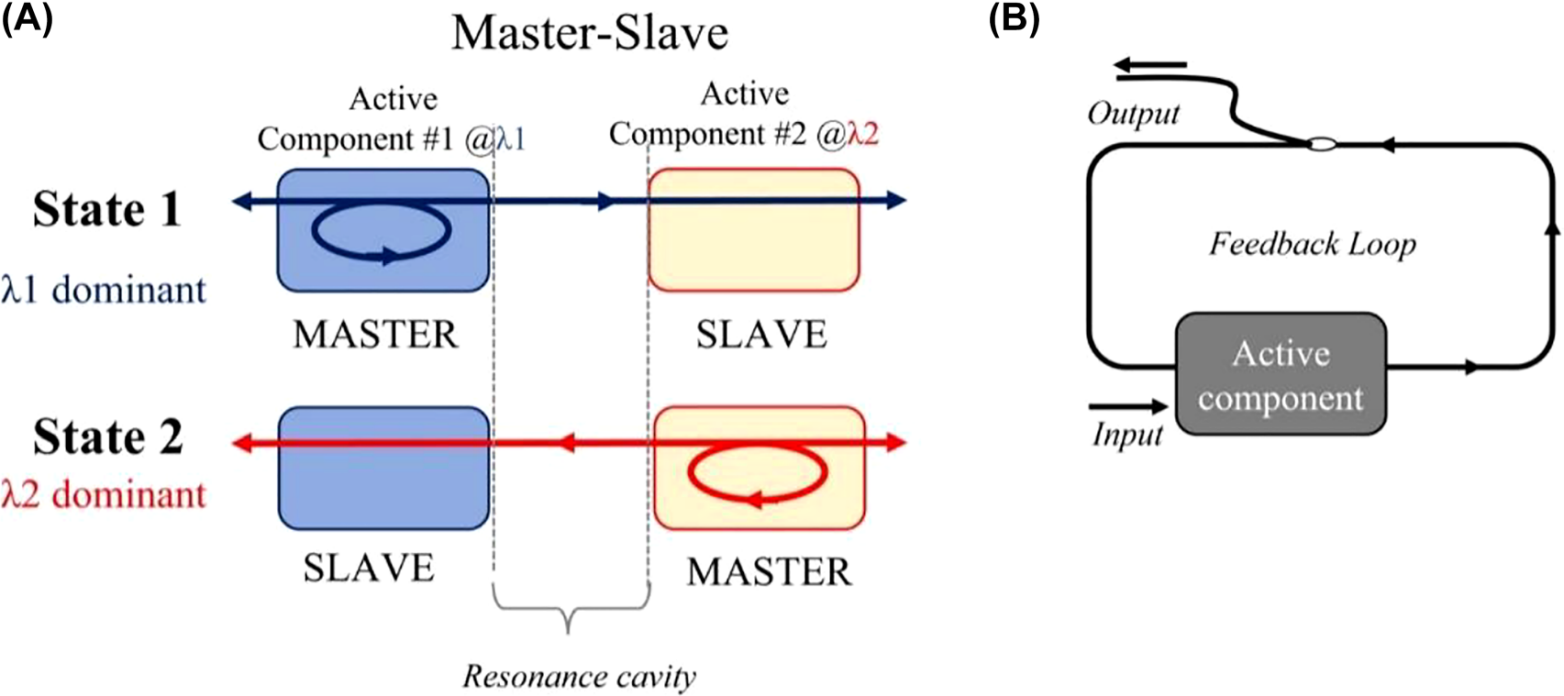
Optical memory with SOA-based bistability based on: (A) master-slave configuration, (B) feedback loop scheme.

Optical memories that use semiconductor optical amplifier (SOA)-based configurations have been pursued during recent years mainly due to their increased integration maturity. As such, optical memories relying on the bistability of engineered optical resonances created between SOAs or SOA-based Mach–Zehnder Interferometric (MZI) structures have been widely exploited following master–slave configuration [93–99]. As shown in Figure 7A, two active components that are usually either SOAs or SOA-MZIs are placed in a cross-coupled arrangement forming an artificial cavity. The discrete memory states (i.e., the logical value “1” or logical value “0”) are represented by two different wavelengths emitted by the respective active component. Each time, only one of the two available wavelengths can be dominant in the cavity, whereas at the same time, the other wavelength remains suppressed. Assuming that *λ*_1_ and *λ*_2_ wavelengths refer to the logical value of “1” and “0” respectively emitted by active component #1, then State 1 corresponds to the cavity state where the light at *λ*_1_ dominates the cavity and suppresses wavelength *λ*_2_ emitted by active component #2. As long as State 1 lasts, active component #1 acts as the “master” while active component #2 serves as the “slave” and the memory output signal is imprinted at *λ*_1_ wavelength. On the contrary, in State 2, the wavelength at *λ*_2_ suppresses wavelength *λ*_1_, and the memory output emits a signal at *λ*_2_ wavelength. The alternation between the two logical states is achieved by injecting external light at the appropriate amount of power and wavelength to the “master” component, suppressing its operation and allowing the “slave” component to recover back to its equilibrium state. In this case, the wavelength emitted by the “slave” device can then reach the “master” device acting as a holding signal that maintains the suppression of the former “master” wavelength even if the external light injection stops. This type of configuration is usually employed for set–reset flip flops (SR-FFs) that have also been used in optical SRAM cells [93, 94, 96, 99]. Theoretical studies on cross-coupled schemes [94, 96] have highlighted that the switching time between the two states is inversely proportional to the cavity length formed between the two active components, suggesting that an integrated solution must be adopted to enable switching times at a picosecond scale.

Optical memories with SOA-MZIs configured on feedback loops have also been demonstrated [95, 97]. As depicted in Figure 7B, this scheme requires a single active component (i.e. an SOA-MZI switch) and an external cavity usually implemented by loop configurations that feed the output signal back to the active element utilizing fiber or an integrated bent waveguide [95]. In fact, the artificial cavity based on the loop serves as the memory element enabling bit storage while a tap of the cavity is usually exploited to monitor the memory content. The active element employed so far relied on a 1×2 optical SOA-MZI switch that either feeds the loop with the switched signal securing the continuity of its recirculation, or blocks the recirculation by switching the signal out of the loop. This type of flip–flop memory has been demonstrated in set–reset operational schemes [93, 94, 96, 99] using independent and discrete set and reset externally injected signals and has also been employed to build toggle flip–flops (T-FFs) [95, 97] by applying a single external pulsed signal.

#### 2.2.2 Semiconductor and charge trapping devices

**Figure 8:**
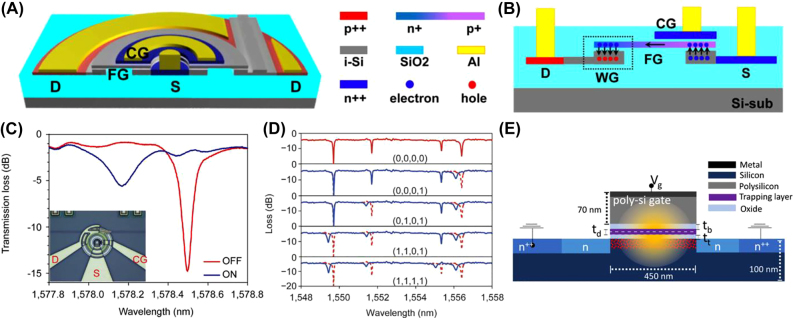
Optical memories based on trapped charge effects. (A–D) A CMOS-compatible, electrically programable, and optically readable memory device [103]. (A) Tilted and (B) cross-sectional schematic of the photonic memory cell. The black arrows show the flow of charge carriers during program and erase operations. (C) Transmission spectra when the memory cell is “ON” and “OFF”. There is a 325 pm blue-shift in the resonant wavelength when the device is turned “ON”. (D) Transmission spectra of the microring memory array. Each microring can be individually programed. (E) Cross-sectional schematic of the SAHAS structure [104].

Barrios and Lipson first proposed a CMOS-compatible optical memory device utilizing the dispersion effect induced by charge accumulation in a floating gate [100]. Since then, many studies have attempted to simulate and experimentally demonstrate the feasibility of the proposed concept [101]. Song et al. fabricated an electrically programable photonic memory cell based on a microring resonator using standard CMOS-compatible technology [102, 103]. The device structure and the operation principle are shown in Figure 8A and B. To program the cell, the control gate is positively biased to induce an electric field between the source and the control gate. Once the field is sufficiently high, electrons are injected into the floating gate and redistributed due to the self-generated electric field of the p-n junction. The accumulation of charge carriers at the drain side reduces the refractive index and causes a blue shift in the microring’s resonant wavelength, as shown in Figure 8C. This switches the device to its “ON” state. To erase, the drain terminal is biased to extract the electrons from the floating gate. This again modulates the refractive index of the floating gate, resetting the resonant wavelength to the original value, and switches the device to its “OFF” state. The device was also studied for multilevel programmability by varying the input pulse width. Once a resonance shift is induced, the memory state remains stable without bias for up to 20 h. Additionally, four microring resonators with slightly varying radii are coupled to a single bus waveguide to form a cascaded array. The difference in resonant wavelengths enables 4-bit simultaneous optical readout as shown in Figure 8D.

A recent work simulated a SAHAS (silicon–aluminum oxide-hafnium aluminum oxide) charge trapping structure that shows a decrease in the writing/erasing voltages and an increase in switching speed [104]. HfO_2_ is chosen as the charge storage layer due to its high trapping density, while Al_2_O_3_ is selected as the surrounding oxide layer due to its high dielectric constant. Using this structure shown in Figure 8E, the authors hope to increase the overlap between the guided mode and the trapped charges to enhance light–matter interaction.

#### 2.2.3 Tunable photonic crystals

**Figure 9:**
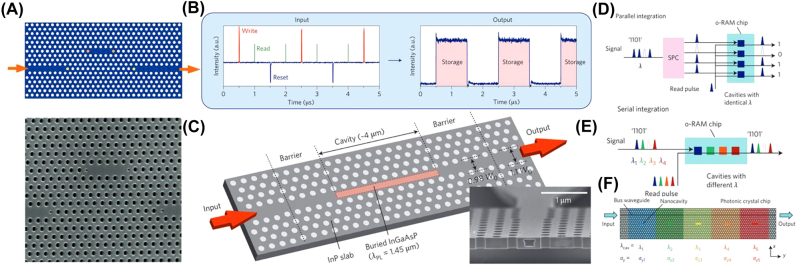
Tunable photonic crystals. (A) Schematic (Top) and SEM image (Bottom) of the photonic crystal nanocavity [105]. (B) Operational principle of the all-optical RAM showing two full cycles of write, storage, and erase operations. Input waveform (left) is consisted of write, read, and reset pulses superimposed on the continuous wavelength bias [108]. (C) Schematic of the InGaAsP buried heterostructure in InP-based photonic crystal. The inset shows the cross-sectional SEM image of the fabricated structure [108]. (D–E) Conceptual illustration for parallel (D) and serial (E) integration of PhC devices. (F) Nanocavities with different resonant wavelengths that are side-coupled are also coupled to a common bus waveguide, forming a cascaded configuration [109].

All-optical switching and memory operations can be achieved by coupling a nanocavity that acts as a bistable switch to a photonic crystal (PhC) waveguide [105–107]. Figure 9A and B shows the design and the operation principle of this memory device. A continuous wavelength bias light that is detuned from the resonant wavelength is initially launched into the cavity; this results in low output intensity. A writing pulse is then sent into the device, which significantly reduces the refractive index of the cavity due to optically generated charge carriers; this causes a blue shift in the resonant wavelength that matches the original detune of the bias wavelength. The bias light can now enter the cavity, and the output intensity shifts to the higher level or the “on” state. Finally, a reset pulse is applied. It depletes the accumulated charge carriers in the cavity returning the refractive index to the original value. This brings the device back to the lower output level, or the “off” state, and a complete switching cycle is demonstrated [108].

However, this type of memory device does require a constant optical bias above the bistability threshold, making it volatile. The continuous optical bias dominates the energy consumption and significantly increases the total power consumption. Additionally, during the carrier generation process, heating causes a red shift of the resonant wavelength. This cancels out with the intended blue shift, further diminishing the device performance. Many studies attempted to address these problems [110], [111], [112] until Nozaki et al. suggested a novel buried heterostructure design that seemed most promising [110], [111], [112]. By embedding a bulk InGaAsP active region into an InP photonic crystal line defect (Figure 9C), both carriers and photons are strongly confined within the nanocavity while maintaining a high thermal conductivity [108]. With these modifications to the nanocavity, average power consumption as low as 30 nW and a memory holding time greater than 10s were achieved. Four-bit memory was also demonstrated by integrating devices in a parallel array, schematically shown in Figure 9D.

The same group later demonstrated the scalability of their design by establishing a serial integration instead of the parallel array configuration [109]. Nanocavities with different resonant wavelengths were integrated serially while sharing the same bus waveguide (Figure 9E and F). This way, packaging density can be increased, and the WDM capability can be fully exploited.

### 2.3 Ferroelectric (BTO)

The Pockels effect is a linear electro-optic phenomenon in materials with non-centrosymmetric crystal structure [113]. The refractive index of these materials can be modified by tuning the Pockels coefficient and is proportional to the strength of an applied electric field. Examples of conventional materials that exhibit the Pockels effect include lithium niobate (LN), lead zirconate titanate (PZY), and aluminium nitride. LN with a Pockels coefficient of ∼30 pm V^−1^ was believed to be the most suitable material for silicon photonics integration until Abel et al. reported their findings on barium titanate crystals (BTO). The Pockels coefficient of BTO film is ∼923 pm V^−1^, 30 times that of LN [114].

**Figure 10:**
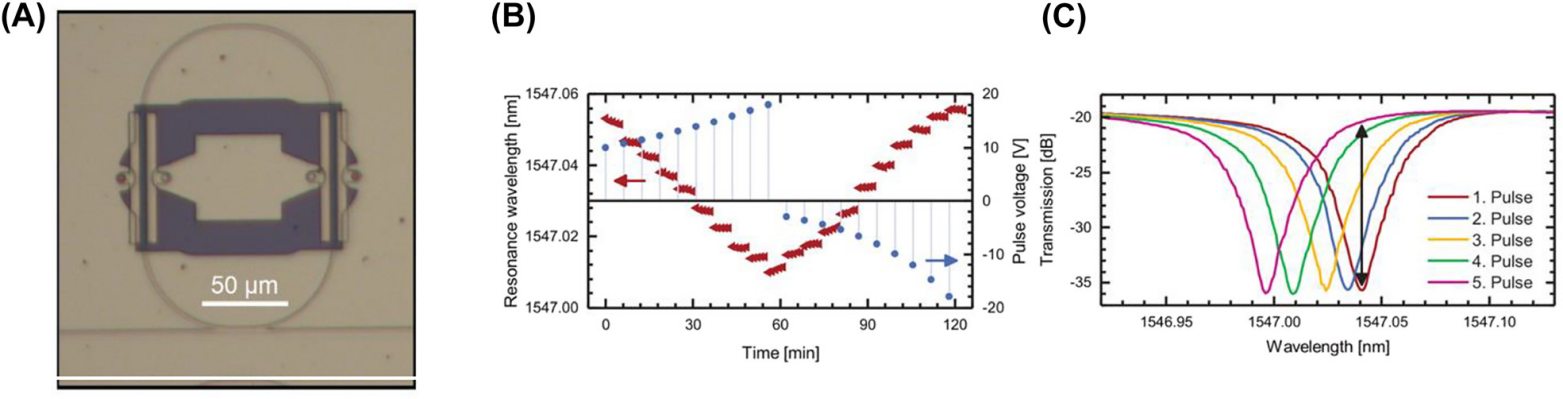
Ferroelectric barium titanate phase shifter in integrated photonic circuits. (A) BTO incorporated into a racetrack resonator [117]. (B) Voltage pulses between −10 V and +20 V are applied to set the device to 10 distinguishable resonant wavelengths. (C) Transmission spectrum after five voltage pulses are applied with the transmission contrast labeled [115].

Initially, the ferroelectric material has randomly distributed domains. After poling, all domains are polarized in the same direction, saturating the electro-optical response in the ferroelectric thin-film. This will act as a reference point before sweeping the bias voltage. When a voltage pulse opposite to the poling bias is applied, it switches a fraction of the domains to the opposing ferroelectric polarization. By applying pulse voltages between −10 V and +20 V, Abel et al. showed that the resonance wavelength of BTO-based racetrack resonators (Figure 10A) could be shifted by a few picometers (Figure 10C) [115, 116]. Transmission contrast at a fixed wavelength can exceed 15 dB, and a minimum of 10 non-volatile transmission states was achieved, indicating the feasibility of multi-level memory (Figure 10B). Device performance was further improved by Geler-Kremer et al. as they were able to set over 100 states in a single device [117].

Recent findings have also indicated the scalability of BaTiO_3_ on silicon photonic platforms [118]. Eltes et al. have successfully transferred BTO thin films grown on 200 mm SOI substrate to a silicon wafer. A variety of thin-film characterizations were performed, including XRD and ellipsometry, to confirm the high homogeneity and quality of the transferred film. Device performance was evaluated using both passive ring resonators and active MZMs. A propagation loss of 5.8 dB/cm was extracted from ring resonators. This value can be further improved by minimizing scattering losses in the Si waveguides. Using an unbalanced MZM, the DC $V_{\pi }$ L value extracted to be 0.23 V cm. Along with the extremely low absorption coefficient of BTO, $\alpha V_{\pi }$ L was calculated to be 1.3 VdB. This value is an order of magnitude smaller than any available high-speed Si modulator. As indicated by Geler-Kremer et al., the Pockels effect in BTO only modulates the real part of its refractive index [117]. This means that BTO can only act as a phase shifter and must be integrated with racetrack resonators or a device with optical interference to achieve multilevel, amplitude-modulated memory. Additionally, the change in the refractive index of BTO is linearly related to the applied electric field. Thus, although the ferroelectric domain orientation is nonvolatile and can be used as a photonic memory based on phase shifts, the readout can only be done when an external electric field is applied.

### 2.4 Optomechanical memories

**Figure 11:**
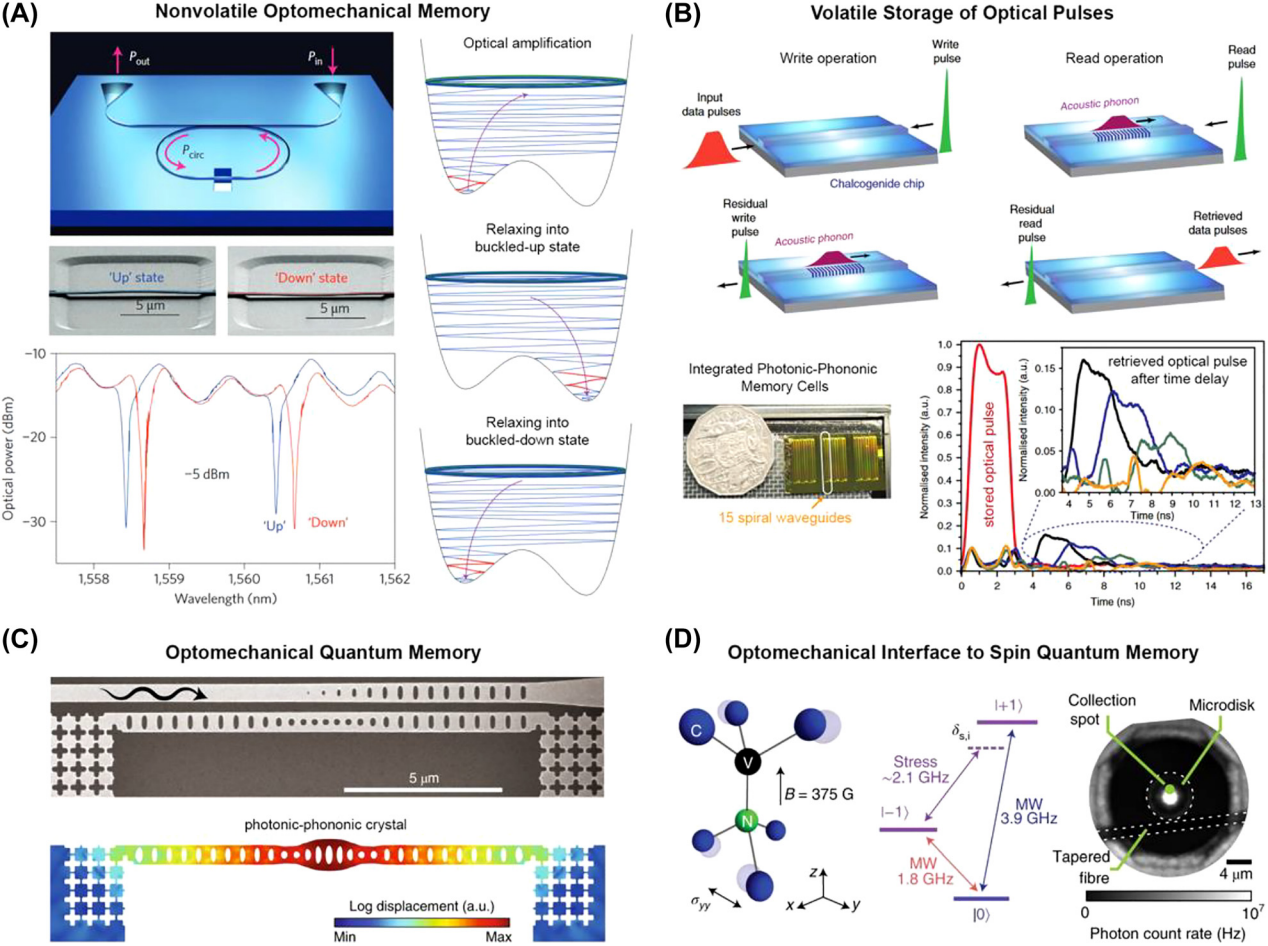
Optomechanical methods for all-optical storage of classical and quantum information. (A) Demonstration of nonvolatile optical memory using a partially suspended ring resonator exhibiting a mechanical bistability [125]. (B) Volatile storage of optical pulses using stimulated Brillouin scattering in chalcogenide spiral waveguides [122]. (C) SEM image and simulated mechanical displacement of an optomechanical quantum memory cell addressable at telecommunication wavelengths [128]. (D) Use of mechanical modes in a diamond microdisk resonator to manipulate spin quantum memory states [129].

The coupling of light to phonons (i.e., “optomechanics”) is another powerful approach for the storage and manipulation of optical information [119, 120]. The use of phonons as a coupling mechanism between light and matter has enabled volatile storage of optical pulses through Brillouin scattering in cm-long fibers and waveguides [121–123] (Figure 11B) as well as in nanoscale cavity-based devices [124]. Mechanical devices can also store optical information in a non-volatile manner by using light to manipulate mechanically bistable nanoscale structures coupled to a photonic waveguide or resonator. For example, Bagheri et al. [125] demonstrated all-optical control of a microring resonator containing a suspended waveguide with two stable mechanical configurations – either “buckled up” or “buckled down” (see Figure 11A). The displacement of the waveguide tuned the optical resonance of the cavity through interaction with the substrate, causing a change in transmission. By optically exciting mechanical resonance of the nanobeam [126] and then actively cooling through optomechanical damping, it was shown that the final state of the resonator (i.e., the “up” or “down” state) could be chosen at will. The field of optomechanics is rapidly evolving, and now it is also possible to exploit mechanical bistability in MEMS-enabled photonics to achieve nonvolatile photonic memory through either optical attenuation or a phase shift [127].

While optomechanics for optical storage has seen significant research efforts over the past few decades, the approaches mentioned above require relatively complex storage and retrieval strategies. They typically have volatile responses on the order of tens of nanoseconds (or are limited in write speed by the mechanical response of the resonator in question in the case of bi-stable mechanical resonators). However, photon-phonon coupling remains a powerful interface between the optical domain and various physical phenomena. Recently, photon-phonon coupling has been used to store and manipulate quantum memory [128, 129]. These unique approaches can enable the nanoscale engineering of mechanical qubits with long coherence times [128] (Figure 11C) or provide an alternative to controlling various spin-based qubits which may not be directly accessible to optical transitions [129] (Figure 11D).

### 2.5 Other optoelectronic effects

While not memory in the strictest sense, any device which modulates an optical signal using an external stimulus can be used as volatile memory in a photonic computing architecture. This includes electro-optic phase shifters embedded in resonators and interferometers employing effects such as the thermo-optic [130, 131], electro-optic [114, 132, 133], plasma-dispersion [134, 135], piezoelectric [136, 137], and photorefractive effects [138, 139]. The physical movement of waveguides through micro-electromechanical systems (MEMS) [140, 141] is another high-efficiency modulation approach that has been used in emerging photonic computing architectures [142, 143]. An advantage of using such modulators as reconfigurable photonic memory cells is their relative maturity and foundry compatibility compared to many other memory technologies outlined above. However, volatility, insertion loss, modulator footprint, speed, and energy consumption (especially for thermo-optic approaches) can be a downside and need to be carefully considered when using modulator designs for photonic memory arrays and computing architectures.

## 3 Photonic memory array architectures

Several memory devices have been proposed using the above-mentioned physical mechanisms. Yet very few of them have demonstrated seamless integration to scalable devices and systems for large volume storage. This section introduces some notable advances in scalable photonic integrated memory array architectures.

### 3.1 Memory cell architectures

Memory elements form the primary units for storing the data information in computing systems. Depending on the memory accessing and processing time of the stored data, there have been three main conventional electronic memory cell devices: the static RAM cells, the dynamic RAM (DRAM) cells, and the CAM cell architectures [4, 144].

**Figure 12:**
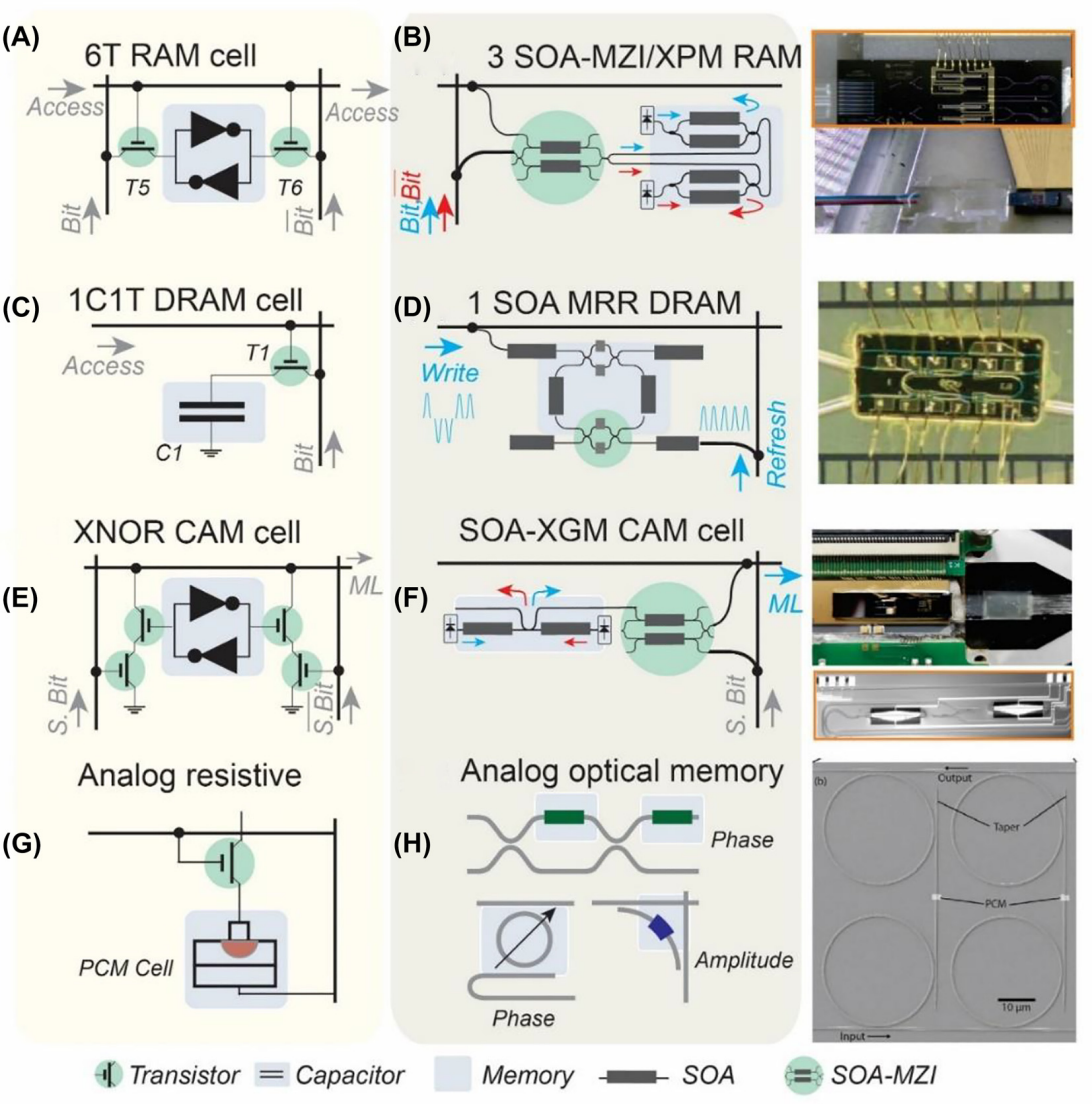
Comparison of electronic and photonic SRAM, DRAM, CAM, and analog cell architectures: (A) conventional electronic 6T RAM cell architecture, (B) integrated photonic RAM cell architecture based on three SOA-MZI XPM gates and inset-photo of a fabricated device [145], (C) conventional electronic DRAM cell architecture with 1capacitor and 1 transistor, (D) integrated photonic DRAM cell architecture and inset-photo of a fabricated device [146], (E) architecture of a conventional electronic NOR CAM cell and (F) integrated photonic CAM cell architecture based on three SOA XGM gates and inset-photo of a fabricated device. (G) analog resistive memory based on phase-change materials for in-memory computing. (H) Photonic integrated circuits based on active phase and amplitude modulators and inset of a PCM-based photonic cell array [147].

Figure 12A depicts the architecture of a standard electronic six transistor (6T) static RAM cell architecture [4]. The SRAM cell comprises two CMOS inverters cross-coupled together back-to-back, as indicated with a grey-colored highlight, where the output of the first inverter is fed as input to the second inverter and vice versa. This cross-coupled setup forms a bistable device configuration-layout that acts as the main storage unit of one of the two possible logical states (“0” or “1”). To change the logical state of the storage unit, two external data signals *Bit* and $\bar{Bit}$ are provided to the inputs of the cross-coupled inverters, configuring the circuitry to the respective logical state. Moreover, random access to the memory cell is granted by two additional transistors, as marked with a green color highlight in Figure 12A, one per input port of the cross-coupled inverters, acting as on/off gating elements that allow or block access to the external data signals to the storage unit.

Stemming from the well-known ‘Memory Wall’ bottleneck, optical random-access memories (RAMs) have been proposed as high bandwidth variants, with the goal of performing all equivalent RAM operations directly in the optical domain. The first demonstration of an all-optical RAM cell was presented in [93], adopting the direct analogous layout of the electronic SRAM cell and replacing each circuit component with the photonic alternative. Specifically, the storage unit was implemented on a hybrid photonic integrated circuit based on two cross-coupled semiconductor optical amplifier – Mach–Zehnder interferometer (SOA-MZI), where the unswitched output of the first SOA-MZI is fed as a control signal to the second SOA-MZI, setting in the switched operating condition; hence, implementing the photonic flip–flop memory equivalent of the electronic memory unit with the two cross-coupled inverters. Specifically, exploiting cross phase modulation phenomena (XPM), one of the two SOA-MZIs acts as master switch, suppressing the opposite SOA-MZI that hence acts as a slave switch, while the role of the master and slave SOA-MZI switches can be interchanged due to the symmetrical setup, allowing to represent the logical “1” and a logical “0”. Switching between the two logical states, and thus, writing a logical “0” or “1” requires injecting proper external SR pulses at the control branch of the master SOA-MZI, as described for the bistable photonic memory devices in Section 2.2.1. On the other hand, random access to the control branches of the two SOA-MZIs is granted by two SOA cross-gain Modulation (XGM) switches that act as On/Off gating elements, i.e. one per data-input branch equivalently to T5 and T6 transistors of the electronic SRAM, allowing or blocking the external data signals to each optical storage unit. Following the direct analogous of the electronic SRAM, with one AG per input branch, a similar optical RAM cell was demonstrated in [148], performing at 1 Gb/s based on two integrated SOA-XGM On/Off gates and an SOA-based ring laser fabricated on a multiple quantum well (MWQ) AlGaInAs/InP wafer.

However, towards exploiting the optical property of wavelength diversity of light [96], the external optical data signals of the RAM cell (*Bit*, $\bar{Bit}$ and *Access*) can be transmitted at different wavelengths. This allows multiplexing the three signals in one WDM data stream, propagating simultaneously through a single input port to the optical RAM cell, towards reducing the number of active elements used for granting random access. Specifically, the proposed optical RAM cell layout is schematically represented in Figure 12B, necessitating only one On/Off AG shared between the *Bit* and $\bar{Bit}$ data signals. Such an all optical WDM RAM cell architecture operating at 5 Gb/s was presented in [99], utilizing a monolithically integrated InP FF memory and a single SOA-MZI AG that exploits the wavelength diversity of the data signals. Using a single broadband multiwavelength AG, rather than two, to simultaneously control the on–off operation of the data signals allows reducing the number of active elements. A photo of the packaged InP memory device is also depicted in the inset of Figure 12B. Moreover, the InP FF memory occupied an area of 6 × 2 mm^2^ and a length of 5 mm for the coupling waveguide between the two SOA-MZI switches, being 5× smaller than the respective hybrid integrated FF memory [93]. The proposed optical RAM cell was later demonstrated using a large set of different memory-controlling signals at various wavelength pairs from 1534 up to 1560 nm. The results revealed clear eye diagrams with error-free operation across the whole C-band, indicating that it can be utilized as a generic RAM unit in the columns and rows of two-dimensional memory matrices. Finally, by operating the SOA-MZI AG in a deeply saturated gain condition and a differentially-biased push-pull scheme, it allowed circumventing the limited carrier lifetime of the SOA-gain recovery, providing a much faster response time down to 25ps for the AG. Thus, achieving all RAM cell functionalities (namely *Read*, *Write* and *Grant Access*) at 10 Gb/s [145] and well exceeding the speed of previous fast electronic RAMs [149]. Although most of the reported developments have focused on the use of bistable SOA-based photonic devices for optical RAM cell implementations, in principle, any of the previously described memory devices of Section 2 can be deployed in equivalent optical RAM cell devices. For instance, following the principles of the presented WDM optical RAM cell, more sophisticated integrated photonics technologies relying on III-V-on-SOI PhC have been experimentally shown to support 10 Gb/s operation, either as AG On/Off units or FF memory operations [150]. Such memory featured a compact footprint of 6.2 μm^2^ and power consumption in the order of 3.2 fJ/bit, raising the expectations to start competing with electronic RAM counterparts.

On the other hand, the layout of a conventional electronic DRAM cell is depicted in Figure 12C, consisting of a single capacitor connected in series with an additional transistor. The role of the capacitor is to remain charged or discharged, acting as the main memory unit that stores the logical ‘1’ or ‘0’, while the transistor acts as the AG that grants access to the stored memory content, enabling or disabling it to reach the DRAM output. Although the electronic DRAM cell features a simple circuit layout, its carriers are being discharged and not permanently retained, requiring a frequent refresh. In analogy to the electronic DRAM cells that are widely deployed in the computing domain, optical DRAM cell can integrate optical cavity-resonators and optical feedback loop architectures with high Q-factors as bistable optical memory units of a recirculating light, along with a periodic optical pulse signal that can refresh the stored recirculating optical data-information [146, 151]. Although a fiber-based implementation of a dynamic RAM cell was presented in [151], an integrated photonic DRAM cell fabricated on an InP-InGaAsP material platform was recently demonstrated [146]. The layout of the integrated photonic DRAM cell is shown in Figure 12D, comprising an SOA-based integrated microring loop and an MZI gating unit, while a photo of the fabricated device is shown in the inset of Figure 12D. Specifically, the photonic DRAM cell relies on six SOAs integrated into an active ring resonator that is optically refreshable, allowing to retain a recirculating optical pulse for a certain fraction of time. An external optical pulse needs to be optically injected inside the loop in phase with the memory pulse signal to extend its memory retention time. An out-of-phase optical pulse signal can be injected into the loop to erase the stored signal. The integrated photonic DRAM cell was demonstrated at a 2.5 GHz writing speed and instant optical read-out through a secondary optical tap-output and a memory retention time as long as 100.5 ns.

The third type of conventional memory-cell architecture forms the CAM cells. CAM cells are typically employed for data-intensive and content-based data operations such as fast look-up memory tables, as described in the next section. In its most straightforward architecture, conventional electronic CAM cells rely on the XNOR layout [144], which is schematically depicted in Figure 12E. In particular, it comprises an FF memory unit, e.g. relying on the cross-coupled inverter scheme as marked with grey color, for storing the logical data, i.e. a “1” or “0”, and an XNOR logical gate that performs a logical-comparison between the stored bit and the incoming search bit. The XNOR logical gate is formed by the additional transistor elements marked with green color and acts as two pull-down paths. These are fed with the signal of an input search bit and the stored data-signal of the FF, while the result of the XNOR comparison operation is transmitted at the matchline branch. Specifically, when there is a mismatch between the input search bit and the stored bit, i.e. an XOR logic operation, then a short-circuit between the Matchline and the ground is implemented, activating a pull-down path that discharges the Matchline signal and resulting in a logical “0” transited at the CAM cell output. On the other hand, when the search bit and stored data match, i.e. an XNOR logic operation, the matchline is not discharged to the ground but instead is propagated to the CAN cell output, indicating a logical “1”.

The photonic alternative of the conventional electronic CAM cell is depicted in Figure 12F. Equivalently to the electronic cell architecture, it consists of an optical memory unit that stores the desired data value and an optical logic gate for comparison purposes. The optical FF unit, as marked with grey color in Figure 12F, can, in principle, rely on any integrated photonic memory unit. The first demonstration of an optical CAM cell architecture [152] exploited a coupled SOA-MZI integrated FF and an external fiber pigtailed SOA-MZI acting as the comparison logic gate. In contrast, a complete CAM cell unit was recently presented in [153], relying on a bistable photonic waveguide FF memory of distinctive simplicity that monolithically integrates two coupled SOA-XGM travelling waveguides on a generic InP platform. A photonic of the packaged InP integrated CAM cell is shown in the inset of Figure 12F. The optical XOR logic gate relies on an all-optical SOA-MZI switch powered by a CW laser source at its input. The two control branches of the SOA-MZI are reach fed with the Search Bit and the output of the FF, respectively, allowing to perform an all-optical XOR comparison operation that is emitted at the Switched port of the SOA-MZI, while being experimentally demonstrated to support speeds up to 10 Gb/s [152].

When considering optical versions of electrical analog memory cells (Figure 12G), there are many approaches to achieving tunable optical attenuation on-chip. MZIs and microring resonators with integrated phase shifters have both been used as programmable memory banks for various computational architectures [154, 155]. PCMs on both crossbar arrays [156, 157] and arrays of microring resonators (SEM image of two unit cells is shown in Figure 12H) [147] can also serve as a wavelength-addressable method to address 2D arrays of multilevel optical memory cells. While the motivation for these architectures is primarily intended for machine learning acceleration or neuromorphic computing applications [158] (see Section 4 for further discussion), these approaches can also be used for high-speed optical access to nonvolatile multi-bit data storage.

### 3.2 Addressing multicell arrays with look up tables

The development of the basic photonic memory cells such as the RAM, DRAM, CAM, and analog memory aimed to formulate the basic building blocks for building scalable and more practical optical memory applications. In this direction, the first step towards scaling to larger capacity memory architecture is scaling to multi-bit one-dimensional (1D) memory lines, which requires identifying the complete optical memory line interconnect architecture. An indicative 1D memory-line architecture is the Matchline architecture of Address Look-Up (AL) memories found in packet-switches and routers.

**Figure 13:**
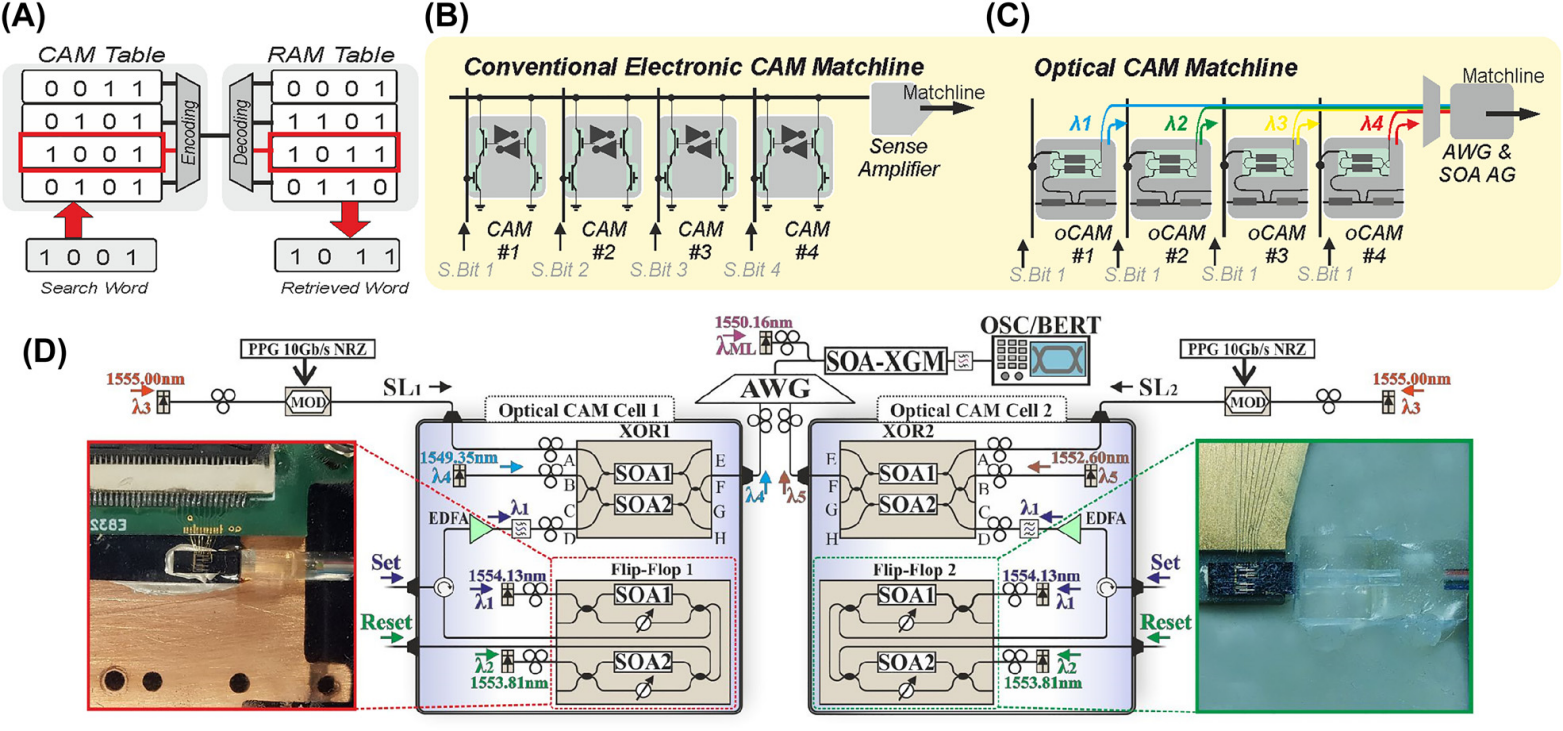
Multicell arrays with look up tables. (A) Schematic diagram of a look-up memory table architecture. (B) Layout of conventional 4-bit electronic CAM Matchline layout. (C) Layout of a 4-bit optical CAM Matchline layout. (D) Experimental implementation of the first optical 2-bit CAM Matchine computing with photonic memories.

With the increasing optical linerates, routers and packet-switches need to resolve the output port of an incoming packet effectively and within a reasonable latency and power-consumption envelope. AL functionalities are typically performed in multi-bit memory-lines of CAM cells [144]. The schematic layout of a CAM Matchline is shown in Figure 13A, being interconnected to a RAM line. The CAM line stores router’s destination addresses, and the RAM line the respective output port. In this configuration, the destination address of an incoming packet, termed in Figure 13A, as “search word”, is compared with the stored bits of each CAM cell of the CAM Matchline for a fast bitwise comparison. In case a match is found, and all logical values of the stored-bits and destination-address bits are equal, a read operation of the interconnected RAM row is performed, and the output port of the router-path is obtained.

Figure 13A shows an example of a stored path “1 0 0 1” being equal to the destination address, and hence activating the path “1 0 1 1” stored within the RAM line. The circuit layout of a conventional 4-bit electronic CAM Matchline is shown in Figure 13B. As described in the previous sections, it consists of four CAM cells whose outputs are coupled together to the same electrical wire, interconnected to a sense amplifier (SA). The role of the SA is to generate a respective signal that shows whether a match between the destination address and the stored path has been obtained. The conceptual circuit layout of the photonic alternative of a 4-bit ML architecture is shown in Figure 13C.

The all-optical CAM Matchline can perform the storage and search operations directly in the optical domain. Each CAM cell comprises an optical flip–flop memory unit for storing a single bit and an XOR gate as a comparison gate, as presented in Figure 12F. The output of each CAM cell can then be imprinted at a different wavelength *λ*_*i*_, with a zero-power level at the output of the XOR memory-comparison operation corresponding to a matching case. This operation allows the four outputs of the 4-bit optical CAM Matchline to be wavelength-multiplexed in a single photonic optical line, through an optical multiplexer such as an AWG.

The multiplexed 4-bit optical Matchline signal will then be a multilevel signal, with each power level representing the number of miss-matches within the CAM cells of the Matchline. Hence, a zero-power level can be obtained only when all four XOR outputs of the Matchline emit a logical “0” value that corresponds to a complete match within the memory row. The role of the electronic SA can then be replaced by a simple SOA wavelength converter, acting as a simple On/Off gating unit power by a CW wavelength. In this way, any non-zero signal of the 4 four CAM cells will saturate the gain of the SOA, blocking the CW from reaching the output and setting it in the off state. On the contrary, when all four CAM cells feature a zero-power level, the CW will reach the SOA-output, operating in the on state, and imprinting a logical “1” at the final Matchline output, to indicate a complete match. The first experimental demonstration of such a 2-bit all-optical Matchline was recently presented in [159], as shown at the inset in Figure 13D. It is also worth noting that alternative optoelectronic Matchline architectures have also been proposed using cascaded ring-resonators driven by electrical memory output signals [160], while large scale WDM photonic memory line architectures have been demonstrated with up to 100-bit capacities using photonic crystals [109].

## 4 Computing with photonic memories

Computing with photonic memories can be done in the digital or the analog domain. In the former, binary memories suffice, and their function is limited to store 0’s or 1’s. For the analog domain, the so-called computational memories are required. These are analog memories with multilevel or continuous tunability response, which allows to simultaneously store and perform arithmetic and/or logic operations in the same memory cell. This section explores architectures for computing with photonic memories in both the digital and analog domains. While an in-detail comparison of performance metrics between electronic and photonic computing architectures is not part of this review’s scope, relevant analysis in this topic can be found in Nahmias et al. [161] and Li et al. [162].

### 4.1 Digital

**Figure 14:**
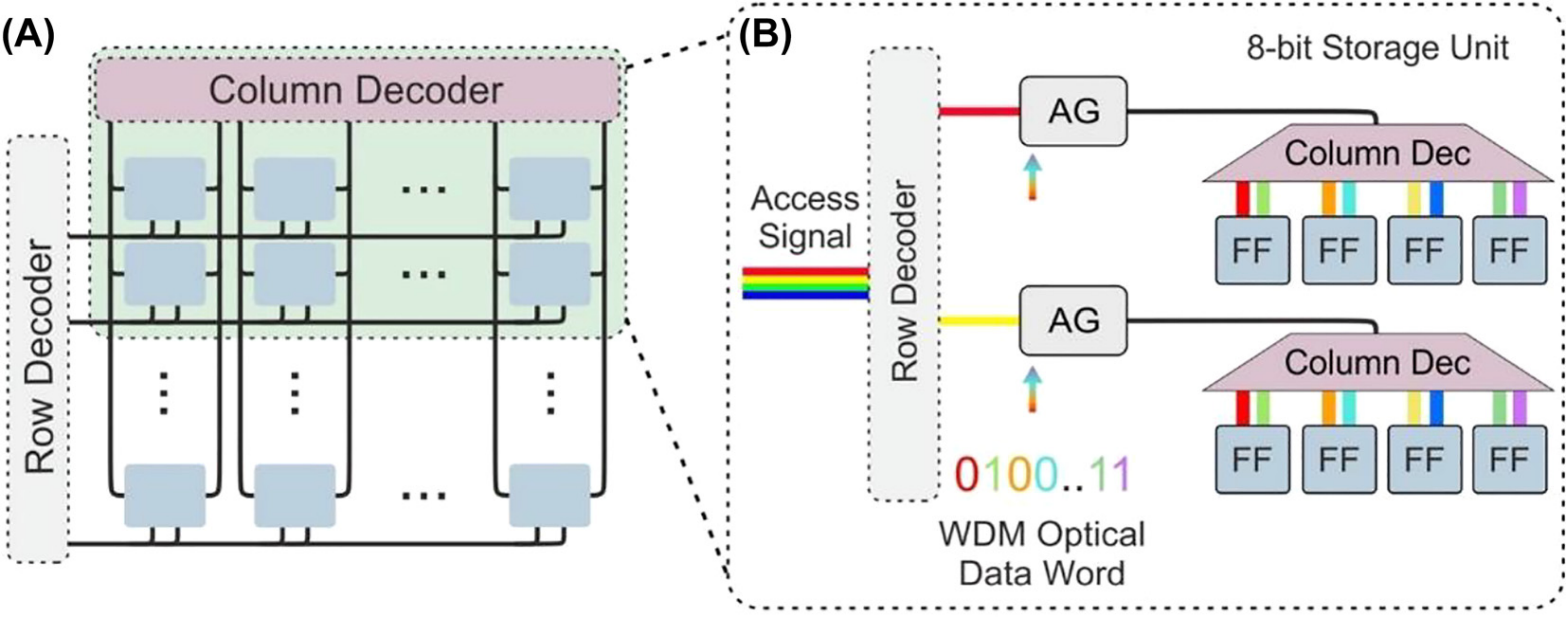
Computing with digital photonic memories. (A) Concept of a 2D 4 × 4 optical RAM bank and (B) 16-bit optical storage unit with a single row decoder unit and simultaneous operation in both rows with a passive column decoder in each row.

A typical two-dimensional (2D) arrangement of 4 × 4 static RAM bank can be seen in Figure 14A, where 16 separate single RAM cells are organized in four rows and four columns and the selection of each desired row/column is performed by Row/Column Decoder circuits, respectively. The row decoder circuit operates with a single Address Line (AL) signal that can be a single- or a multi-wavelength stream, exploiting WDM technology. The number of rows accessed in the RAM bank is given by 2^*n*^, with *n* being the number of wavelengths incorporating the AL. In the above case, each row can store a 4-bit data word, which is defined by the number of columns, while each cell can be accessed by the column decoder. All four rows are operating simultaneously, and based on the logical value of the Access signal shared among the RAM cells of a single row, simultaneous access for Read or Write operation is either allowed or blocked in the row. Exploiting the wavelength dimension that is inherently offered by light has proven beneficial in the past as it (a) can provide wavelength diversity in a single all-optical RAM cell [96] (b) can minimize the total number of active AG elements in multicell configurations to only one AG per row powered by a multi-wavelength input word stream [163, 164] and (c) allows complete passive decoding by processing WDM-formatted words [93].

The proposed scheme of the 16-bit RAM storage, as illustrated in Figure 14B, benefits from utilizing the WDM technique by employing a single multi-*λ* AG per row and a single passive column decoder (CD), while the same WDM-data word stream is injected in both AGs, providing also the capability to reuse the wavelengths in each distinctive row. The proposed 16-bit RAM storage architecture concept comprises four multiwavelength AGs, each corresponding to a row of four all-optical memory cells operating with wavelength diversity in the bit inputs. A 4-bit WDM-formatted optical data word encoded in eight different wavelengths, representing the complementary bit and signals, is transmitted simultaneously at the four AGs, and a different access signal (AS) is propagated to its designated AG through the RD. In this scenario, only one AG will allow the incoming 4-bit WDM-formatted data word to propagate to the four all-optical flip–flop memory cells with a CD, enabling a completely passive column decoding using a passive AWG, while the second AG will block any access to the memory-cells. Each memory cell requires two wavelengths to power up CW1/CW2, which are reusable for all flip–flops, and each row will store the 8-wavelength WDM data word. During the Write functionality, the Access bit of the desired row unblocks the corresponding AG and allows writing of the incoming 8 wavelengths coded 4-bit data word in a specific row. For the Read operation, the Access bit signal will permit only the contents of the corresponding row to emerge as the output. Moreover, the decoding procedure is performed entirely passively as both Write and Read functionality share the same WDM-based circuitry [163].

A nonvolatile photonic memory for digital computing can use phase change memories with binary operation, a functionality demonstrated both all-optically and electro-optically, as discussed in Section 2.1.1. An experimental bank of all-photonic PCM memories was notably demonstrated by Feldmann et al. [156], where a 16×16-pixel greyscale image was stored in the optical domain using 2-bit PCM memory cells and a 2D microring resonator array for a total storage of 512 optical bits. Von-Neumann architectures requiring long-term data retention have been proposed using optically-controlled memory banks based PCMs [165, 166]. In general, computer architectures bringing closer processing and memory units can benefit from optical memory banks that alleviate the data movement bottleneck, especially if the communication is done with optical waveguides following the efforts to integrate photonics with silicon nanoelectronics [14].

### 4.2 In-memory computing

The design of modern computer systems is based on the von Neumann architecture, where processing is performed in the central process unit (CPU) while the results are stored separately in memory [28]. With the significant increase in CPU processing speed, memory improvements have relied heavily on storage density. The latency of data transfer between the CPU and memory is limiting the speed of operation and this is known as the von Neumann bottleneck [167, 168]. Any approach to overcome the von Neumann bottleneck requires a significant change to the current computer architecture, and an example of such is in-memory computing. Computing in-memory essentially combines processing and memory to happen in the same device, which requires a memory capable of storing multiple levels of information to carry analog computing tasks such as scalar multiplication.

#### 4.2.1 Phase change computational memories

**Figure 15:**
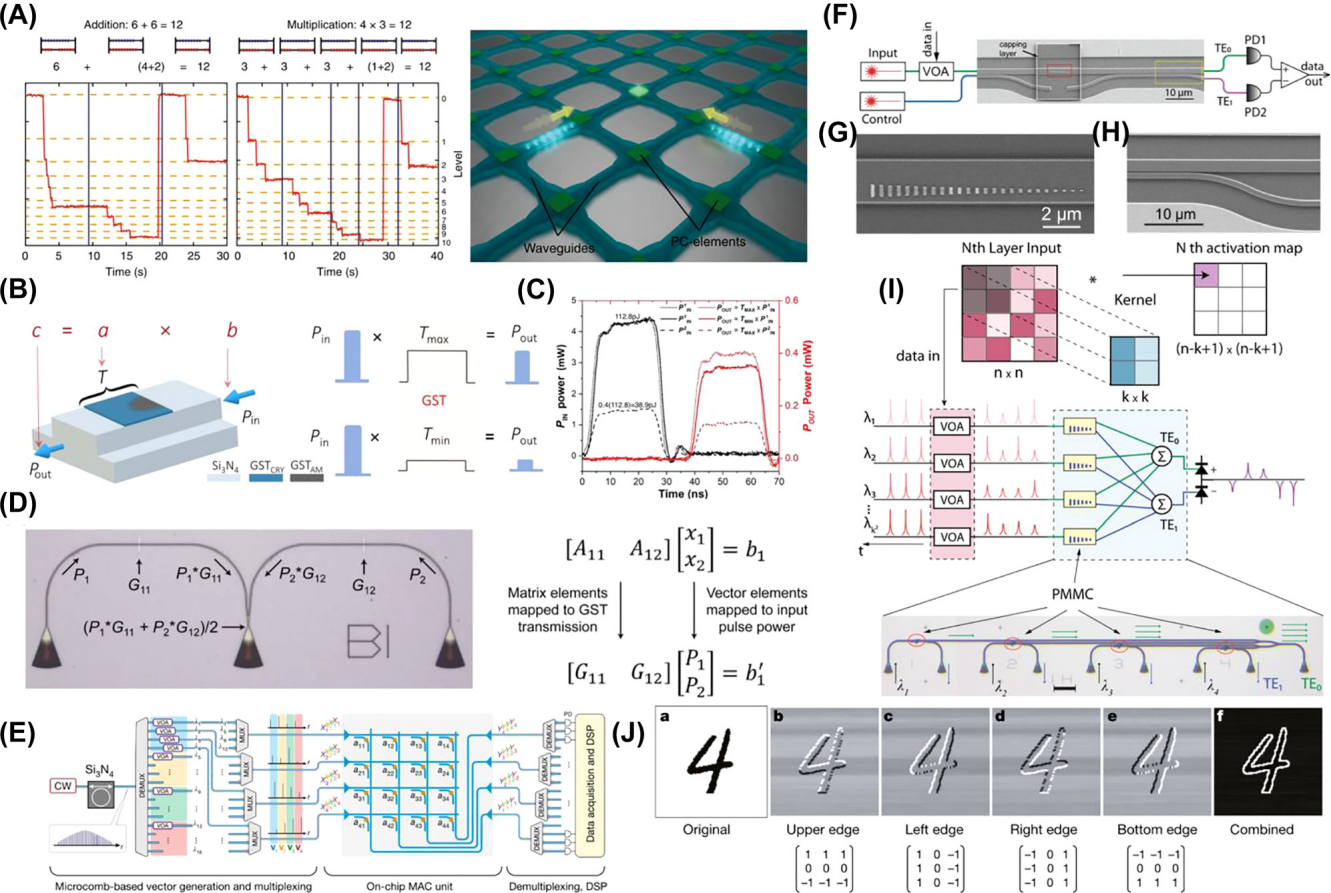
In-memory computing using PCMs. (A) An on-chip all optical abacus [168]. Left: A base-10 addition of ‘ 6+6=12 ’ is calculated. Middle: Multiplication of ‘ 3×4=12 ’, equivalent to sequential addition, is calculated. Right: The waveguide crossing array. PCMs are placed at every crossing point and can only be switched when two overlapping pulses are applied. (B–D) Demonstration of direct scalar multiplication and matrix–vector multiplication on a photonic platform [169]. (B) By mapping the multiplicand ‘a’ to transmittance (*T*) and the multiplier ‘b’ to input power (*P*_in_), answer ‘c’ can be obtained by measuring the output power (*P*_out_) as $P_{\text{out}}=T\left(P_{\text{write}}\right)\times P_{\text{in}}$ . (C) Real-time measurement of the input and output pulses for three multiplication operations. (D) Optical image of the circuit design to implement matrix–vector multiplication. (E) and (J) An integrated photonic tensor core consisted of phase-change memory cells to perform convolutional image processing [157]. (E) Conceptual illustration of an architecture that incorporates the photonic tensor core to compute convolutional operations. (J) Four image kernels of size 3×3 each highlighting a different edge of the original image. (F–I) A programmable phase-gradient metasurface mode converter (PMMC) that is scalable to realize an optical convolutional neural network (OCNN) [55]. (F) SEM image of the PMMC and the corresponding setup for device programing and measurement. (G) Zoomed-in SEM image to show the geometry of the GST nano-antenna. (H) The mode selector that can couple the TE_1_ mode out of the bus waveguide. (I) Operation principle for convolutional image processing using a PMMC array. Kernels of dimensions k×k are encoded into $k^{2}$ number of PMMCs. Pixels of the input image are grouped into ${\left(n-k+1\right)}^{2}$ patches that are encoded as optical pulses.

As explained in Section 2.1.1, PCMs possess a “natural accumulation property”, meaning their current state depends on prior excitation history. Feldmann et al. took advantage of this property to perform basic arithmetic operations using an all-photonic abacus [169]. An example of this was the demonstration of adding ‘6 + 6’, as shown in Figure 15A. Six pulse sequences, representing the first summand, are sent in to crystallize the PCM-cell from level zero to level six, and each pulse sequence consists of five consecutive 12 pJ pulses. Subsequently, pulse sequences that are equal to the second summand are sent into the PCM-cell. Once the cell reaches the tenth level, or fully crystalline, a sequence of ten 19 pJ reset pulses is sent in to reamorphize the cell. Meanwhile, a carryover is performed onto a second PCM-cell to store the tenth digit information. Finally, the remaining pulse sequences are applied. This series of operations should leave the first cell at level two and the second cell at level one, presenting the answer of ‘12’. Multiplication of ‘ 4×3=12 ’ was also computed similarly by successive addition.

Nonetheless, it is easily noticeable that sequential addition is inefficient for multiplying two numbers in terms of switching energy and computational speed. Rios et al. offered potential solutions by performing direct scalar and matrix–vector multiplications using PCM based photonic elements shown in Figures 15B and D, respectively [170]. Precisely, the direct scalar multiplication of a×b=c , with a,b,c∈[0,1] , was accomplished by mapping the multiplicand *a* to the transmittance (*T*) of the GST cell and the multiplier *b* to the input pulse power (*P*_in_). First, a Write (amorphization) pulse (*P*_Write_) is used to program the cell to a specific transmission state. Then, a second pulse with energy $E_{P_{\text{In}}}<E_{P_{\text{Write}}}$ , insufficient to alter the phase configuration of GST, is sent into the device and will experience the amplitude modulation induced by the transmittance of GST. Finally, the output power (*P*_Out_) is measured, providing the answer to this multiplication. To Erase or crystallize the cell, either a sequence of decreasing-energy pulses or a single two-step pulse can be used. The former can bring the device to any partially crystallized states, while the latter can get the cell back to the baseline or fully crystalline state. Three operations, 0×1=0 , 1×1=1 , and 1×0.4=0.4 , are demonstrated in Figure 15C using the maximum (*T*_MAX_) and minimum (*T*_MIN_) transmission and maximum ( $P_{\text{in}}^{1}$ ) and intermediate ( $P_{\text{in}}^{2}$ ) input pulse energy.

These memory cells’ nonvolatile nature and reconfigurability can support matrix–vector multiplications when a suitable architecture is used. By implementing the photonic circuitry shown in Figure 15D, the result of each multiplication performed at the two memory cells is combined using a beam splitter. The 1×2 matrix $\left[G_{11}\;G_{12}\right]$ is preprogramed into the two cells’ transmittance and multiplied by a 2×1 vector contained in a pair of input pulses *P*_1_ and *P*_2_. Additionally, the authors also proposed using multiple cascaded power splitters and wavelength multiplexing to increase the computational density in these devices.

Given these prior efforts to carry out in-memory computing on a photonic platform, many studies have proposed and demonstrated architectures to bridge the gap between isolated computing devices and form large-scale optical networks [55, 157, 171, 172]. Feldmann et al. constructed an integrated photonic hardware accelerator (tensor core) that can operate at speeds of 10^12^ multiply-accumulate operations (MAC) per second [157]. Figure 15E shows the operational concept of the photonic tensor core. The key to this approach is to encode input data into the amplitude and wavelengths of the input broadband source (frequency comb). The input vectors are then grouped using wavelength multiplexing technology and sent into a 2D array of on-chip MAC units based on PCMs, where computations are performed. Once the multiplications are completed, the resultant vectors are demultiplexed and retrieved by photodetectors. To experimentally demonstrate this concept, the photonic tensor core was used to process an image “4” for edge detection. Four kernels, each highlighting a different edge of the original image, are shown in Figure 15J, and the combined image shows that all edges have been successfully detected. Overall, this approach is highly parallel, fast, scalable, and efficient paving the way for optical systems to compete with electronics in data-heavy AI applications.

Another prototypical optical convolutional neural network (OCNN) was established by Wu et al. based on programable on-waveguide metasurfaces using PCMs [55]. Figure 15G schematically shows this phase-change metasurface mode converter (PMMC) directly integrated on a SiN waveguide. Depending on the geometry of the nano-antennae, a well-defined phase gradient dΦ/dx is introduced to the light propagating in the waveguide. The metasurface is designed to only induce the phase gradient in the cGST phase so that the cGST metasurface can convert the fundamental TE_0_ mode into the first-order TE_1_ mode. The phase gradient is much diminished in the aGST phase and is insufficient to support the mode conversion. Right below the multimode waveguide is a single-mode coupling waveguide, and together, they act as a mode selector enlarged in Figure 15H. The TE_1_ mode component will be coupled out of the multimode waveguide while the TE_0_ remains in the multimode waveguide. The output powers of both modes are then measured, and up to 64 levels in modal contrast were achieved. A network of four PMMCs was used for prototyping the OCNN. Convolutional edge detection using this PMMC core was experimentally demonstrated in Figure 15I.

### 4.3 Neuromorphic computing

In the brain, synapses can sense, store, and process data in the same place. This is the same goal with photonic computational memories towards synaptic devices. This section explores the application of photonic computational memories with accumulation or threshold switching behavior to perform nonlinear operations, i.e. mimic the synapse response in integrate-and-fire spiking neurons.

#### 4.3.1 Phase change synapses

Owing to their strong nonlinear and nonvolatile response to light, current, and heat, phase change materials are highly promising analog memories for synaptic connections. Crucially, both the amorphous and crystalline states can stability coexist in the same nanoscale volume of material, allowing continuous tuning of the optoelectronic properties of the memory cell through the application of multiple optical or electrical pulses. This pulse accumulation effect has been widely explored in the electrical domain to create 2D arrays of synaptic connections [173, 174] as well as integrate-and-fire neurons [175]. While synaptic plasticity and correlation detection has been demonstrated in individual photonic devices [176, 177], system-level neuromorphic photonic platforms have been proposed [178, 179], yet remained an outstanding challenge.

**Figure 16:**
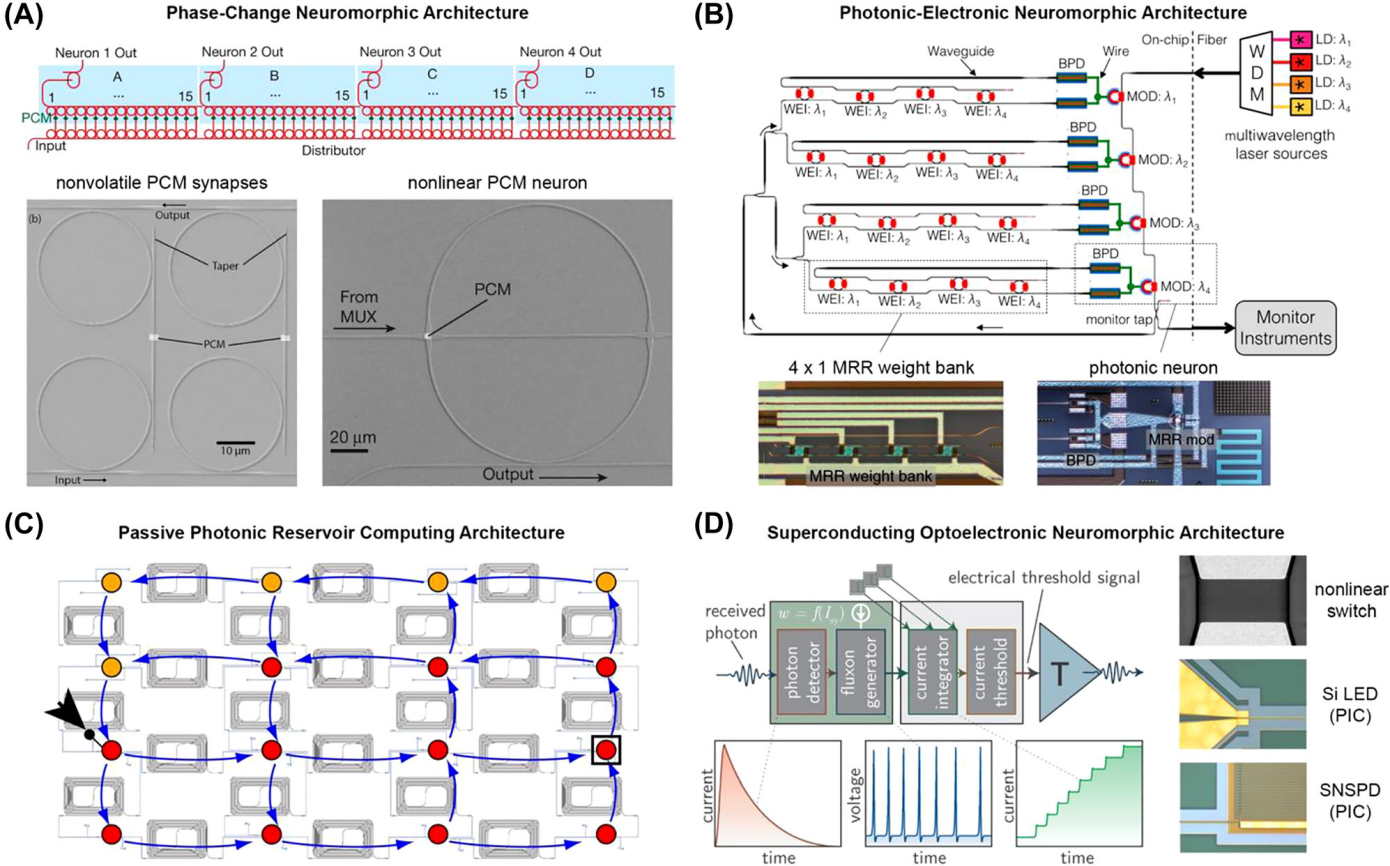
Photonic integrated neuromorphic computing architectures. (A) Neuromorphic architecture which exploits the nonvolatile and nonlinear nature of phase-change materials to implement both synaptic weights and neuron activation functions [147, 171]. (B) Foundry-compatible neuromorphic architecture which leverages optical–electrical–optical (O–E–O) conversion to implement fan-in and nonlinear activations. Add-drop microring resonator banks are used for synaptic weighting [182, 183]. (C) Fully passive photonic reservoir computing architecture using optical feedback and delay lines to implement fading short-term memory [184]. (D) Superconducting optoelectronic architecture for ultralow energy neuromorphic computing [185]. The superconducting nonlinear switch provides the voltage gain needed to drive silicon LEDs which are coupled to superconducting nanowire single photon detectors (SNSPD) via a photonic integrated circuit (PIC) [186].

In 2019, Feldmann et al. demonstrated a four-neuron, 60-synapse photonic neuromorphic platform capable of both supervised and unsupervised learning [172] (see Figure 16A). In this work, PCMs played a fundamental role in both the nonlinear neuronal response and tunable synaptic weights. In the case of supervised learning, the output neurons were programmed to recognize specific patterns by manually setting the transmission of the PCM synaptic weights. For unsupervised learning, the pulse accumulation effect was used to gradually train the pre-neuron synaptic weights through feedback of the post-neuron output signal during repeated exposure to the desired pattern. The nonlinear response of the PCM to optical pulse amplitude enabled discrimination between the training pattern and noise in the system when the optical signal surpassed the threshold for crystallization. This same nonlinearity was also used to implement the integrate-and-fire optical neurons. Here, multiple inputs were summed using a WDM architecture and integrated using a PCM memory cell integrated in a ring resonator. When the total energy of the summed inputs surpassed the threshold for amorphization, the transmission of the ring resonator increased, allowing the firing of an optical pulse. However, once fired, the neurons required a reset pulse which increases the complexity of the systems. Using nonlinear optoelectronic devices [158] or materials with a volatile response, such as VO-2 [81], saturable absorbers [180, 181], or even GST biased near the crystallization temperature [182], could enable self-resetting optical neurons in future implementations.

#### 4.3.2 Silicon photonic-electronic neuromorphic processing

The same building blocks used to create high-speed integrated optical transceivers [188] and routing fabrics (i.e., silicon-based ring modulators [189], silicon–germanium photodetectors [190], WDM filters [191], etc.) can also be combined to create high-speed optical neurons and reconfigurable synaptic weights [183, 184, 192]. A benefit to this approach is the growing availability of mature, foundry-compatible components which serves to improve yield and enable demonstrations on scalable architectures [158]. An excellent example of this approach is the “broad-cast and weight” architecture (shown in Figure 16B) which uses an array of silicon microring resonators to tune the synaptic weights for optoelectronic neurons [131]. As the optical output intensity is decoupled from the optical inputs through O-E-O conversion via a photodetector, trans-impedence amplifier, and optical modulator, challenges such as nonlinearity, cascadability, and fan-out can all be addressed. Operation speeds are fundamentally limited by the photon time-of-flight in the microring weights, around 50 ps [131]. Real-time signal analysis has been demonstrated using this architecture [184] and significant progress has been made in implementing both high-resolution weight control [193] and nonlinear activation functions [194, 195].

Coherent MZI arrays for matrix–vector multiplication are another approach to achieving the same fan-in synaptic weighting for multiple output neurons [155, 196–198]. Both MZI arrays and microring resonator weight banks implement the same multiply-accumulate functions via matrix–vector multiplication [199]. However, a notable difference between these two architectures is the use of a single wavelength for coherent MZI arrays, compared to the need for multiwavelength sources in the case of incoherent MRR weight banks. Both coherent and incoherent approaches have their relative advantages and disadvantages in terms of tolerance to fabrication variability, availability and efficiency of optical sources, and performance in the limit of low optical powers, which will ultimately determine their scaling limits on-chip [143, 200].

#### 4.3.3 Photonic reservoir computing

The use of optical feedback through photonic delay lines [185, 201, 202] and high-Q resonators [203–205] is an effective method to introduce short-term memory without the need for exotic materials. This approach is particularly well-suited for reservoir computing (RC) architectures which rely on random connectivity between a “reservoir” of neurons to create fading memory through inherent feedback and nonlinear dynamics in the network [206]. Since the network’s memory is due to feedback, RC excels at time-dependent tasks such as speech recognition, forecasting, and compensating for nonlinear distortions in time-varying signals [207]. An RC network is trained by tuning the input and output weights of a subset of neurons from the reservoir, typically through least-square minimization. This architecture has the advantage of being robust to fabrication variability and is simple to train since the reservoir weights are typically chosen from a random distribution and remain fixed.

The high signal bandwidth provided by the optical domain enables ultrafast processing of signals and makes RC networks on an integrated photonic platform highly attractive [208]. This has led to several experimental demonstrations of photonic RC architectures (e.g., fully passive silicon photonic architecture shown in Figure 16C) for applications such as compensating nonlinear distortion in fibers [209] and identifying multibit headers for routing optical signals [185]. Scaling to more complex problems (or longer bit-sequences) requires a larger reservoir with a larger number of connections between neurons to increase the network’s memory capacity and duration – a significant challenge for integrated photonics. This has been addressed by free-space approaches that use three-dimensional space to enable dense connectivity between an extensive array of optical inputs through scattering media [210, 211] or programmable diffractive media such as MEMS mirrors and spatial light modulators [212, 213]. Increasing the connection density through novel integration methods [214, 215] could compete with these free-space approaches and enable scalable photonic RC networks-on-chip.

#### 4.3.4 Superconducting optoelectronic loop neurons

Combining superconducting circuits with optical sources and routing is yet another emerging platform to achieve fast and efficient neuromorphic computing in the optical domain [186]. In this approach, superconducting single-photon detectors (SPDs) are used to detect optical signals at the single-photon level emitted from optical neurons and provide significant signal amplification through nonlinear changes in the conductivity of superconducting nanowires [187, 216] (Figure 16D). Additionally, current flux through superconducting loops can be used for long-term storage of synaptic weights [186]. The current flux in these synaptic weights can then be coupled back to the optical domain by controlling the bias of a transmitter circuit which generates optical pulses via integrated semiconducting lasers. Excitatory synapses bias the transmitter toward the firing threshold, while inhibitory synapses bias the transmitter away from the threshold. The unique combination of superconductors with optical sources at cryogenic temperatures could allow the high optoelectronic gain and fan-out necessary for large-scale, biologically inspired computation.

## 5 Outlook

**Figure 17:**
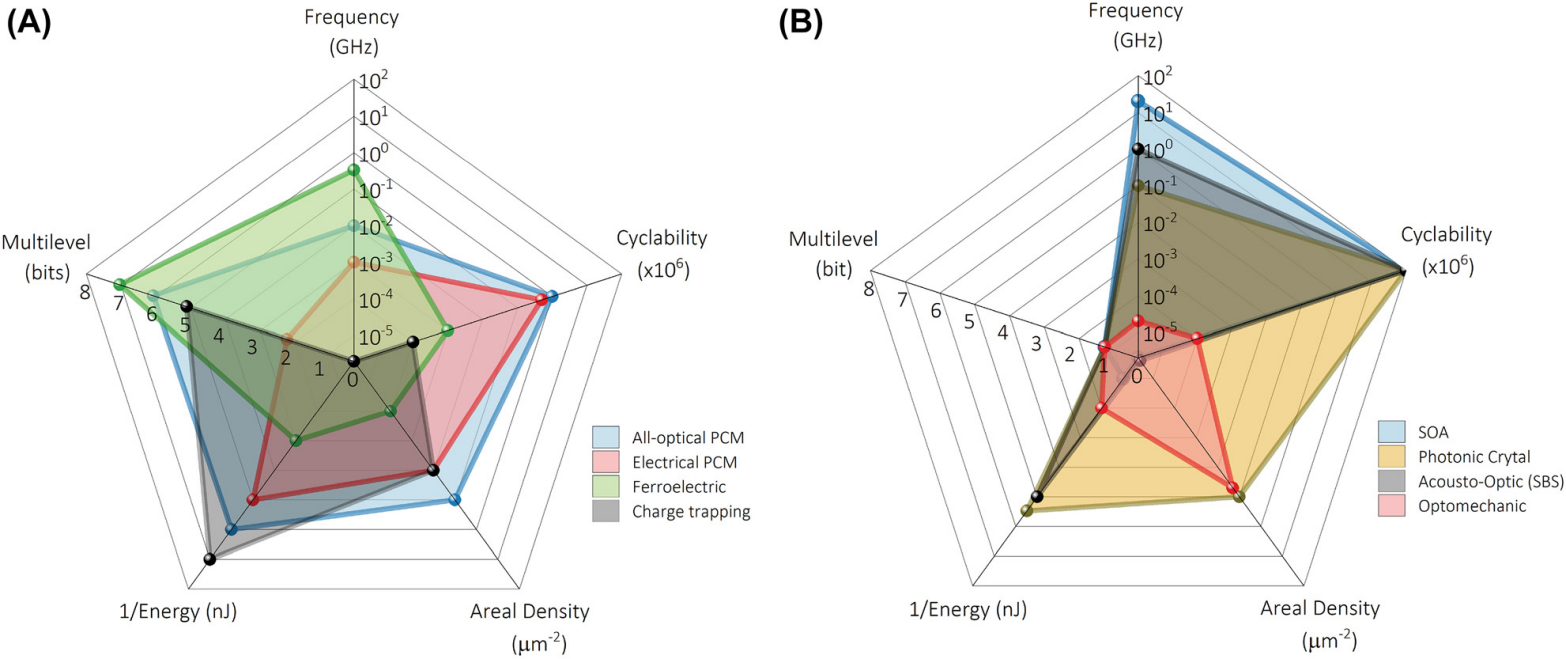
Comparison of photonic memories best-reported performance metrics from Table 1. (A) Nonvolatile and (B) volatile memory cells. The axes in both figures use the same logarithmic scale except for *Multilevel*. *Cyclability* refers to the maximum number of cycles reported for each technology, which might differ from maximum cyclability. For some volatile memories, the underpinning physical mechanism allows one to assume a very large number of cycles, which are usually not reported. For easy visualization, *cyclability* in the latter case takes the maximum value of the axis, yet this does not represent a limit. The value for multilevel is reported for a single memory cell.

The growing list of light-based memories experimentally demonstrated on scalable photonic integrated circuits (summarized in Table 1 and visually compared in Figure 17) clearly indicates the progress and momentum this crucial device has gathered within the field of photonics. Continued research into large-scale integration, novel material platforms, improving fabrication yields, and fully utilizing the inherent bandwidth of photonic in-memory computing could lead to the adoption of photonic memories and processors for specialized applications that are impractical for digital electronics. This includes applications such as high-speed parallel neuromorphic computing for real-time decision making, light-speed and efficient matrix–vector multiplication with multiplexing capabilities for next-generation AI hardware, and ultrafast signal processing for compensating nonlinear distortions in optical networks. Additionally, applications with optical input signals, such as LIDAR, image processing, or fiber-optic telecommunications and computing, would greatly benefit from photonic memories and reduce electro-optical conversions.

**Table 1:** Device performance of different types of memories – PCM-based nonvolatile memories, other nonvolatile memories, bistable memories, and optomechanical memories. Technologies are shaded in grey to represent electro-optical operation and blue to represent all-optical operation.

	Technology	Energy (nJ)^i^	Speed (ns)	Multilevel	Cycling	Footprint ( μ m^2^)^ii^	Application	Limitation
PCM-based nonvolatile memories	W/Ti heater integrated photonic circuit [70]	Write: 0.3 nJ/dB μm ^iii^	>5 × 10^5^	4 × 1-bit	5 × 10^5^	∼100 × 100	Optical signal processing and computing	Large heater footprint
	All-fiber memory device [50]	Set & Reset: 47–54.5	Set: 145	3-bit	>40	∼15 × 15	Storage and logic computing in optical fiber	Complexity in fabrication and scalability
			Reset: 158					
	Waveguide integrated metasurface [55]	Set & Reset: 0.43–3.81	10^6^	6-bit	1000	80 × 20	Convolutional optical neural network	Scaling up to large network is limited by directional coupler efficiency
	PIN diode heater integrated photonic switch [69]	Set: ∼78	10^5^	1-bit	>500	∼200 × 200	Large-scale PCM integration and switching	Low switching speed for recrystallization
		Reset: ∼8						
	P^++^ doped Si resistive heater optical switch [68]	Set: 7 – *n* × 7^iv^	∼100	>2-bit	50	2 × 12	Enables large area PCM integration in silicon photonics	Large heater to PCM area ratio increases power consumption and device footprint
		Reset: 10.4	∼250					
	Single pulse programmable memory cell [54]	Set: 0.68	500	>5-bit	10^6^	∼2 × 5	Neuromorphic computing and in-memory computing	Limited switching area, complex scalability, not suitable for transparent PCMs
		Reset: 68-135	200					
Other nonvolatile memories	Ferroelectric BTO-based memory device [117]	<4 μ W/nm^v^ [116]	300	>6-bit	N/A	∼150 × 100	Phase shifter in neuromorphic and programmable photonic devices	These devices can only act as phase shifters and must be incorporated with other photonic circuits like ring resonators, etc., resulting in an increase in device footprint
	Charge trapping photonic memory device [103]	Erase @10 V: 11.4 pJ	>7.5 × 10^7^	>5-bit	30	∼60	Fabrication fully compatible with CMOS processes	
		Program @6 V: 17.2 pJ	>3.5 × 10^8^					
Bistable memories	Coupled SOA-switches [99]	2 × 10^5^	10^−2^	1-bit	N/A	12 × 10^6^	Optical RAMs and cache memories, optical CAM and address look-up	Large footprint and power consumption
	Buried InGaAsP Photonic Crystal [108]	30 nW (Bias power) + 2.5 fJ (Switching energy)	∼44 × 10^−3^	4 × 1-bit	N/A	∼10	Optical RAMs and logic gates	Requires biasing and stable temperature control
Optomechanical memories	Optical data storage via stimulated Brillouin scattering [216]	<5W^vi^ [217]	14	1-bit	N/A	∼4 × 10^6^	All-optical information processing	Stored information is volatile with a maximum retention time on the order of 10s of ns
	Bistable optomechanical cantilever [125]	10^4^	5 × 10^4^	1-bit	∼20	10 × 10	Nonvolatile optical memory	Ultimate switching speed is limited by mechanical resonance frequency

^i^ A range of programing energy/time is given for devices that are capable of multilevel operation as various energies or pulse durations are required to set the device to the different levels. ^ii^ Unless specifically mentioned by the author, device footprint is a rough estimate based on device images provided in each study. ^iii^ Energy consumption is dependent on the extinction ratio contrast upon GSSe phase transformation and the length of the GSSe cell. ^iv^ Partial crystallization by a single level need 7 nJ of pulse energy. *5-n* number of 7 nJ pulses are required for partial crystallization of the device from the highest level (level 4) to level *n*. ^v^ Power consumption collected from a BTO phase shifter integrated racetrack resonator. The magnitude of the resonance wavelength shift is input power dependent. ^vi^ Energy is not given because the write/read pulses are input pulse width dependent. The power given here was used to write a 200ps data pulse.

The photonic I/O and circuitry components will primarily limit the bandwidth of the photonic memories, and the ability to efficiently multiplex, demultiplex, and detect multiple wavelengths. Therefore, maximizing the bandwidth advantage of photonic memories also requires further development of the passive and active components surrounding them. When employing nonvolatile memories, the multiply-accumulate operations occur at the speed of light and without an energy penalty, where energy is only required to reconfigure the data in memory. However, the energy savings from “free” optical multiplication by simple light propagation are still overshadowed by the laser power consumption and, if performing analog computing, by the digital-to-analog and analog-to-digital conversions [200]. In-detail comparisons of performance metrics centered on computing architectures have been outstandingly elaborated by Nahmias et al. [161], Li et al. [162], and Feldmann et al. [157]. Another significant challenge for photonic memories is the large device footprints, with the waveguide dimensions as a limiting factor. However, even with a photonic memory footprint of ∼1 µm^2^ – over 100 times larger than its electronic counterparts – multiplexing 10 wavelengths and using three layers of waveguides (typical in photonic foundries) would reduce the total storage density disparity from a factor of 100 to ∼3. The storage density can be further improved by multiplexing more wavelengths, employing multilevel storage, or using nanophotonic structures with lower dimensionality than PICs [219]. Alternatively, tight integration between dense electronic memory layers and 3D photonic vias is another path being explored by some groups [16]. Other challenges, such as optical packaging and the integration of exotic materials through advanced manufacturing, still require significant research and development. These limitations, however, are a common challenge for any application of photonic integrated circuits towards a broader technological adoption, which most certainly will see substantial progress in the years to come. While photonic memories still have much to gain from further research and development, their promise of ultrafast readout, multiplexing capabilities, nonlinear response, and ultrahigh bandwidths for analog and neuromorphic computing beyond our current digital processors is highly motivating and would undoubtedly see significant progress in the coming years.
